# Autophagy Protein Atg3 is Essential for Maintaining Mitochondrial Integrity and for Normal Intracellular Development of *Toxoplasma gondii* Tachyzoites

**DOI:** 10.1371/journal.ppat.1002416

**Published:** 2011-12-01

**Authors:** Sébastien Besteiro, Carrie F. Brooks, Boris Striepen, Jean-François Dubremetz

**Affiliations:** 1 UMR 5235 CNRS, Universités de Montpellier 2 et 1, Dynamique des Interactions Membranaires Normales et Pathologiques, Montpellier, France; 2 INSERM, Dynamique des Interactions Membranaires Normales et Pathologiques, Montpellier, France; 3 Center for Tropical and Emerging Global Diseases, University of Georgia, Athens, Georgia, United States of America; University of Michigan, United States of America

## Abstract

Autophagy is a cellular process that is highly conserved among eukaryotes and permits the degradation of cellular material. Autophagy is involved in multiple survival-promoting processes. It not only facilitates the maintenance of cell homeostasis by degrading long-lived proteins and damaged organelles, but it also plays a role in cell differentiation and cell development. Equally important is its function for survival in stress-related conditions such as recycling of proteins and organelles during nutrient starvation. Protozoan parasites have complex life cycles and face dramatically changing environmental conditions; whether autophagy represents a critical coping mechanism throughout these changes remains poorly documented. To investigate this in *Toxoplasma gondii*, we have used TgAtg8 as an autophagosome marker and showed that autophagy and the associated cellular machinery are present and functional in the parasite. In extracellular *T. gondii* tachyzoites, autophagosomes were induced in response to amino acid starvation, but they could also be observed in culture during the normal intracellular development of the parasites. Moreover, we generated a conditional *T. gondii* mutant lacking the orthologue of Atg3, a key autophagy protein. TgAtg3-depleted parasites were unable to regulate the conjugation of TgAtg8 to the autophagosomal membrane. The mutant parasites also exhibited a pronounced fragmentation of their mitochondrion and a drastic growth phenotype. Overall, our results show that TgAtg3-dependent autophagy might be regulating mitochondrial homeostasis during cell division and is essential for the normal development of *T. gondii* tachyzoites.

## Author Summary

Autophagy is a catabolic process involved in maintaining cellular homeostasis in eukaryotic cells, while coping with their changing environmental conditions. Mechanistically, it is also a process of considerable complexity involving multiple protein factors and implying numerous protein-protein and protein-membrane interactions. The cellular material to be degraded by autophagy is contained in a membrane-bound compartment called the autophagosome. We have characterised the formation of autophagosomes in the protozoan parasite *Toxoplasma gondii* by following the relocalisation of autophagosome-bound TgAtg8. Thus, exploiting GFP-TgAtg8 as a marker, we showed that it is a process that is regulated and can be induced artificially by amino acid starvation. Autophagic vesicles were also observed in normally dividing intracellular parasites. Depleting *Toxoplasma* of the TgAtg3 autophagy protein led to an impairment of TgAtg8 conjugation to the autophagosomal membrane and, at the cellular level, to a fragmentation of the single mitochondrion of the parasite and to a severe growth arrest. We have thus found that TgAtg3-dependent autophagy is essential for normal intracellular development of *T. gondii* tachyzoites.

## Introduction

Proteolysis is very important to eukaryotic cells and occurs at a considerable constitutive rate. Base line degradation regulates the levels of numerous proteins and removes misfolded proteins. Mechanistically, this process can be separated into two main pathways: one pathway mediated by the proteasome and the other pathway by the lysosome [1]. The proteasome relies on the ubiquitin system for selection of target proteins and plays a major role in the rapid degradation of short-lived proteins as well as abnormal proteins. The lysosome, which represents the terminal compartment of the endosomal pathway, is a membrane-bound organelle that contains a diverse array of hydrolases for the degradation of plasma membrane proteins and endocytosed extracellular proteins. Lysosomes are also involved in bulk degradation of cytoplasmic components, such as long-lived cytosolic proteins and organelles, and this is achieved through a process called autophagy. Autophagy has been divided into several classes of pathways, such as macroautophagy, microautophagy, and chaperone-mediated autophagy, but macroautophagy has been studied most extensively and we will refer to this specific form of the process as “autophagy” in this manuscript for simplicity.

Autophagy is evolutionarily conserved in eukaryotes from yeast to mammals and has important roles in various cellular functions [2]. The basal role of autophagy is in turnover and recycling of cellular constituents; these housekeeping functions include the elimination of defective proteins and organelles and the prevention of abnormal protein aggregates accumulation. Autophagy also plays an important role in organelles and proteins (but also lipids) recycling under nutrient starvation conditions, as a nutrient source for the cell. Finally, there are pleiotropic and more specialised roles for different eukaryotic cells (in particular in mammals) including cellular remodelling during differentiation and development, regulation of cell longevity and programmed cell death, elimination of invading pathogens and providing antigens to the immune system [2], [3].

In the autophagic process, cytosolic components are sequestered in a double-membrane vesicle known as the autophagosome. The outer membrane of the autophagosome will then fuse with the lysosomal compartment to deliver the inner contents of the vesicle, which will be subsequently degraded. Although autophagy has been initially identified in mammalian cells, the characterisation of the molecular machinery involved in this cellular process has been mainly done in yeast. Due to the ease of genetic analyses in the yeast system, screening for mutants unable to survive nitrogen starvation, allowed the identification of more than thirty *Atg* genes involved in autophagy and the related cytoplasm to vacuole targeting pathway (Cvt) [4]. Of these, some are only present in one organism and others represented by orthologues in different eukaryotic cell types. Among all Atg proteins, Atg8 occupies a central position: it is essential to the process of autophagosome formation, especially for the membrane expansion step [5], [6] and, possibly, the final membrane fusion steps [7]. The protein is present as a soluble form in the cytosol of eukaryotic cells, and gets recruited to the autophagosomal membrane upon induction of autophagy. Interestingly, the binding of Atg8 to the autophagosomal membrane involves two conjugation systems that resemble ubiquitin-conjugation systems [8].

*Toxoplasma gondii* is an obligate intracellular protozoan parasite that is virtually able to infect all species of warm-blooded animals [9]. *T. gondii* is a member of the phylum Apicomplexa, which also includes several other notable human pathogens such as *Plasmodium* and *Cryptosporidium*. The secretory pathway of *T. gondii* is highly polarised and includes several unique organelles devoted to the invasion of the host cell (the micronemes, rhoptries and dense granules) [10]. In addition the parasite harbours the apicoplast, a plastid-like organelle of endosymbiontic origin. The micropore, a cytostome-like structure formed by the invagination of the plasma membrane, can be seen laterally [11], which suggests the existence of an endocytic pathway within the parasite, although it has not been clearly defined at the molecular level. Until very recently, the parasite was thought not to contain any morphological equivalent of “classic” lysosomes, as rhoptries appeared to be the only acidified organelles containing hydrolases in *T. gondii* [12]. Two recent reports have nevertheless identified an acidified vacuole bearing a cathepsin-like peptidase in *Toxoplasma* tachyzoites that could fulfil that role [13], [14], but its precise physiological role remains to be characterised. The autophagic pathway is even less well described in *T. gondii*, although it can reasonably be suspected to be involved in the survival of Apicomplexa, as well as for other parasites with complex life cycles. For example, the importance of autophagy for the development and virulence of protozoan parasites such as the trypanosomatids has been shown previously [15], [16] and recent data have shown an implication of autophagy in the cellular remodelling of *Plasmodium* liver stages [17]. Here, we used the experimental opportunities afforded by *Toxoplasma* as a genetic model organism to investigate the autophagic machinery and assess its physiological role in the parasite.

## Results

### Identification of the autophagic machinery of *T. gondii*

Many of the molecular components involved in autophagy have been identified in the budding yeast *Saccharomyces cerevisiae*. We have thus used the known *S. cerevisiae* Atg protein sequences to identify *T. gondii* homologues through homology searches in the ToxoDB database ([18], Table S1). The core machinery for the assembly of the pre-autophagosomal structure appears to be present in *T. gondii*. This includes the regulating kinase Atg1, as well as proteins central to autophagosome formation itself, like Atg8 and its conjugating partners Atg7–Atg3. In several other eukaryotes, Atg8 requires the activity of two conjugation systems to bind the autophagosomal membrane: Atg7–Atg3 on the one hand and Atg5–Atg12 on the other (see [8], [19] for a review), the latter being dispensable *in vitro* [20].

Interestingly, no clear orthologues of the members of the Atg5–Atg12 conjugation system were found in our homology search. These are also absent from the genomes of several other parasitic protozoa [21]–[23]. In *Leishmania*, distantly related proteins might have similar function [24]. However, the orthologues of these *Leishmania* proteins cannot be found in the *T. gondii* genome. Overall, this either suggests that the Atg5–Atg12 conjugation is not essential for autophagosome formation in *T. gondii*, or that *Toxoplasma* uses different (possibly lineage-specific) proteins to perform this task.

In fact, a significant part of the yeast Atg proteins seems to be missing from the *T. gondii* genome. For instance, most of the Atg proteins involved in the Cvt pathway are not found in *Toxoplasma*. This may not be surprising as this pathway is likely fungi-specific, and is also largely lacking in other eukaryotic systems that are clearly able to perform autophagy [19].

**Figure 1:**
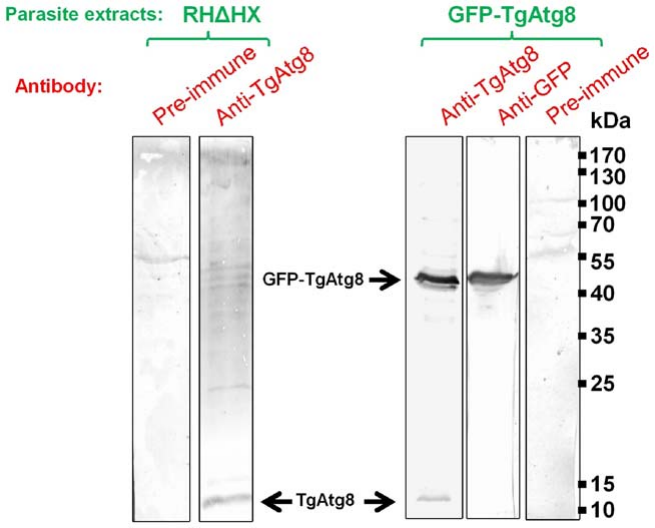
Detection of native and GFP-fused TgAtg8. Protein extracts corresponding to 10^7^ tachyzoites from parental RHΔHX and transgenic GFP-TgAtg8 cell lines were separated by SDS-PAGE and analysed by Western blot using anti-TgAtg8 or anti-GFP antibodies. Overexpressed GFP-TgAtg8 and native TgAtg8 are indicated by arrows.

The most reliable marker for autophagosomes is Atg8 (or LC3, its mammalian counterpart). This protein is essential to autophagosome formation, and stays associated with the autophagosomal membrane from the early membrane recruitment step until the fusion with the lysosomal compartment [25]. Hence, to further characterise the autophagic process, we raised a specific antibody against TgAtg8 (TGME49_054120, Table S1). Using this antibody on tachyzoite lysates, we detected by Western blot analysis a major protein band consistent with the predicted molecular weight of 14 kDa (Figure 1). However, the rather weak signal that was observed suggested a low level of expression for endogenous TgAtg8. To observe the dynamics of autophagosomes formation we then cloned *TgAtg8* in fusion with the green fluorescent protein (GFP) gene to generate a *T. gondii* cell line that stably expresses GFP-TgAtg8 in addition to its endogenous copy. Stable clones of GFP-TgAtg8 transgenic parasites were obtained and showed no apparent difference in growth or morphology (data not shown). When GFP-TgAtg8 parasites were assessed by Western blot with anti-TgAtg8 and anti-GFP antibodies, both detected a fusion protein at the expected molecular weight of 42 kDa (Figure 1). The strong tubulin promoter driving the expression of GFP-TgAtg8 showed an overexpression of ∼20 fold of the GFP-fused TgAtg8 compared with the native protein, which greatly facilitated its detection.

### *T. gondii* is capable of autophagy and the process can be induced by nutrient starvation

**Figure 2:**
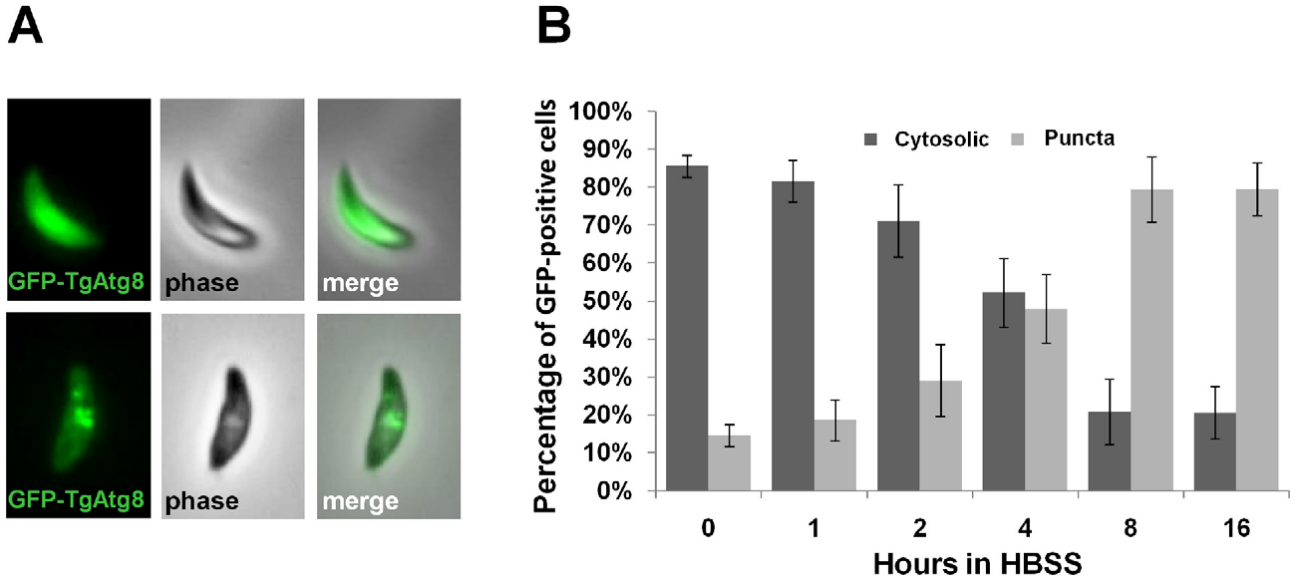
Induction of autophagy by amino acid starvation in extracellular *T. gondii* tachyzoites, assessed by the GFP-TgAtg8 marker. A. Autophagic vesicles marker GFP-TgAtg8 was found in the cytosol of tachyzoites and occasionally in punctate vesicles in recently egressed parasites. B. Extracellular tachyzoites were put to starve in HBSS medium for increased time periods and the proportions of cells displaying punctate or cytosolic GFP-TgAtg8 signals were assessed. Data are mean from *n* = 4 independent experiments ±SEM.

We studied the localisation of the GFP-TgAtg8 fusion protein by fluorescence microscopy on living tachyzoites. In extracellular tachyzoites freshly released from host cells, the GFP-TgAtg8 signal was generally distributed throughout the cytoplasm and also occasionally found in one or more punctate vesicles of various sizes that could correspond to autophagic vesicles (Figure 2A). Using the anti-TgAtg8 antibody, both on the parental cell line and on the GFP-TgAtg8-expressing parasites, we verified by immunofluorescence assay (IFA) that GFP-TgAtg8 and anti-TgAtg8 labelled identical cellular compartments (Figure S1). The GFP-TgAtg8 signal was convenient to follow; we thus conducted our subsequent microscopic observations on the GFP-TgAtg8 cell line. To establish whether the observed structures are autophagic vesicles, we performed starvation experiment on GFP-TgAtg8-expressing extracellular parasites. Free tachyzoites allow a more direct control of environmental conditions and nutrient access, compared with intracellular parasites sheltered within their host cells. Strong induction through starvation is a hallmark of autophagosomes in many eukaryotic systems. To induce starvation, we incubated GFP-TgAtg8-expressing extracellular tachyzoites for increasing intervals of time in an isotonic solution devoid of amino acids (Hank's balanced salt solution, HBSS). We imaged the parasites and quantified our findings. We noticed that the number of parasites bearing GFP-labelled puncta increased over the incubation time (increasing from 15±3% to 79±8% after 8 hours, Figure 2B) as did the number of puncta per parasite (Figure S2A). As a control, incubation of the tachyzoites in complete Dulbecco's modified Eagle medium (DMEM, supplemented with 10% fetal bovine serum) for similar time periods induced no appearance of these puncta (17±2% of parasites bearing GFP-labelled puncta versus 21±2% after 8 hours).

These changes in the GFP-TgAtg8 signal were induced quickly and reached a plateau after ∼8 hours of starvation. The re-localisation of GFP-TgAtg8 from the cytosol to vesicular structures during starvation suggested that these puncta were indeed autophagosomal structures. The presence of punctate GFP-TgAtg8 signal in ∼20% of the extracellular tachyzoites at the start of the experiment might reflect basal autophagy, or a proportion of the population that have egressed early and are already in a relative nutrient stress condition. The majority of these extracellular tachyzoites showed a single relatively large vesicle, which appeared prominently localised in the anterior part of the parasite, in the Golgi apparatus/apicoplast region (Figure S2B). Similarly, we performed microscopic observations on intracellular tachyzoites and found that they also occasionally displayed vesicular GFP-TgAtg8 in normal growth conditions (Figure S2B). Additional vesicles were found to be localised in a non-polarised manner throughout the cytoplasm of the tachyzoites. Although mostly present as a punctate signal, the GFP-positive structures were found to display some heterogeneity as previously described for GFP-TgAtg8 in other eukaryotes [26].

**Figure 3:**
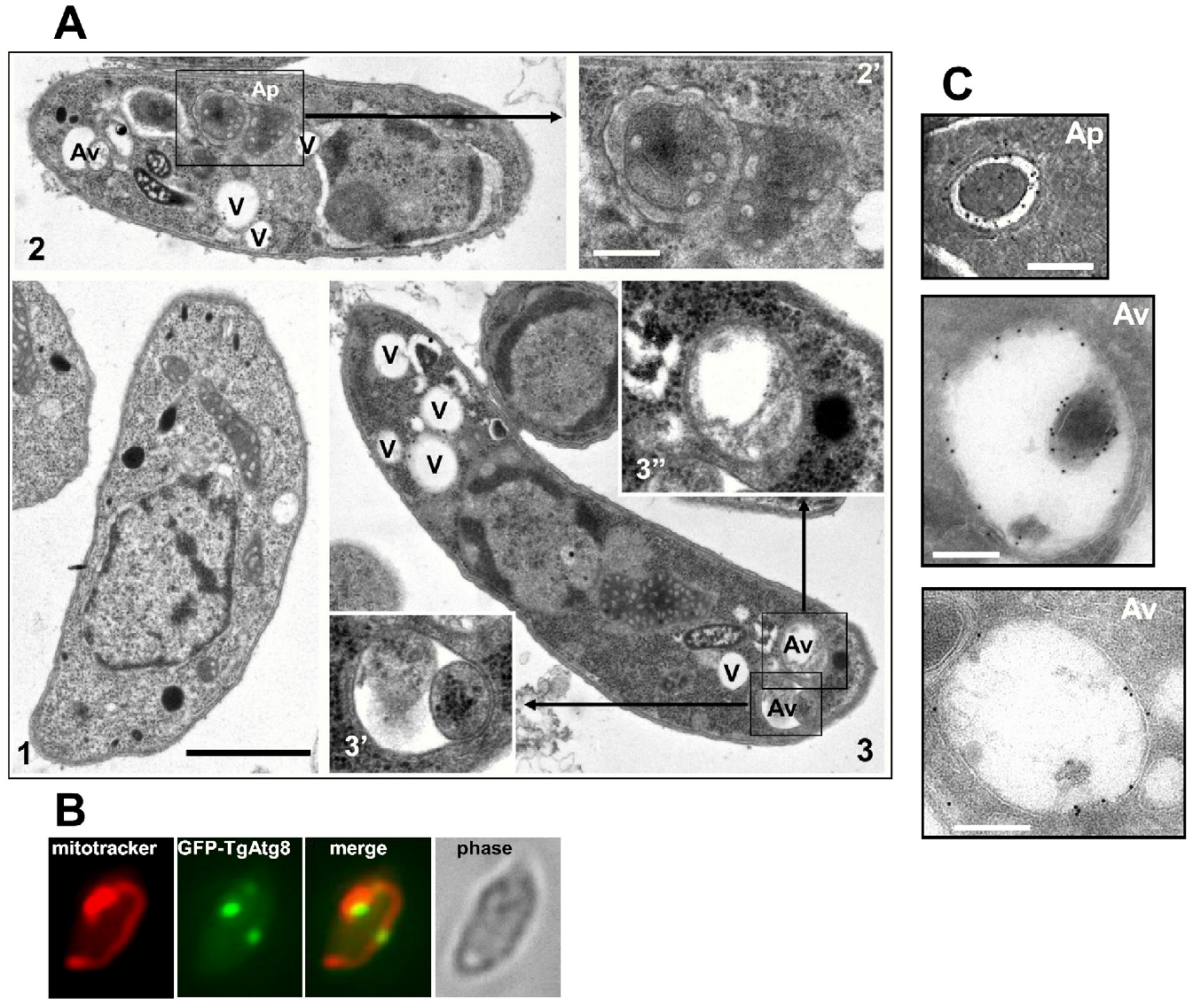
Morphological observation of autophagic vesicles in extracellular tachyzoites by electron microscopy. A. Extracellular tachyzoites were starved for 8 hours in HBSS medium and observed (2, 3). Unstarved control is shown for comparison (1). Several vacuoles (V) can be seen in starved parasites, with also cytosol- and organelle-sequestrating membranous structures resembling autophagosomes (Ap), as well as vacuoles containing partially digested material corresponding to autophagic degradative vacuoles (Av). 2′, 3′ and 3” pictures are a magnification of squared regions. Scale bar = 1 µm in initial micrographs (1, 2, 3) and 0.2 µm in magnifications. B. Partial co-localisation of GFP-TgAtg8-labeled autophagosomes and *Toxoplasma* mitochondrion. Extracellular tachyzoites expressing GFP-TgAtg8 were incubated in complete medium with Mitotracker Red to label the mitochondrion; partial co-localisation could be observed in some parasites displaying autophagic vesicles. C. Identification of autophagic vesicles in extracellular tachyzoites by immuno-electron microscopy. Extracellular tachyzoites expressing GFP-TgAtg8 were starved for 8 hours in HBSS medium, fixed, cryosectioned and stained with an anti-GFP antibody, then with gold-conjugated secondary antibody and observed. Gold particles can be seen around cytosol-sequestrating membranous structures resembling autophagosomes (Ap), as well as larger vacuoles containing partially digested material corresponding to autophagic degradative vacuoles (Av). Scale bar = 200 nm.

We sought to morphologically characterise these autophagic vesicles. Tachyzoites, either freshly released or starved for 8 hours, were fixed and processed for electron microscopy analysis. Compared to the control parasites, the starved tachyzoites displayed a higher number of large cytoplasmic vacuoles (Figure 3A, V). Additionally, in the starved parasites we identified several membrane-bound vesicles of about 300–900 nm in diameter containing cytoplasmic material or organelles. These structures were usually bounded by two membranes (Figure 3, Ap). These are commonly observed structural features of autophagosomes [27]. As an example of organelles that could be found in these vesicles, part of the unique mitochondrion of a tachyzoite was seen segregated inside a membranous compartment (Figure 3A) and co-labelling of GFP-TgAtg8-decorated autophagosomes and mitochondrion showed partial co-localisation (Figure 3B), which is suggestive of mitophagy occurring in *Toxoplasma*. When the autophagosome has fused with the lysosomal compartment to deliver its content for degradation, it becomes an autolysosome, or autophagic vacuole. These degradative vacuoles containing partially degraded cytoplasmic material could also be identified in starved parasites (Figure 3, Av). Importantly, similar structures could be identified in a starved cell line expressing GFP-TgAtg8, by immuno-electron microscopy with an anti-GFP antibody (Figure 3C), providing a link to the observations we made by fluorescence microscopy.

Our results thus show that nutrient starvation triggers the appearance of autophagosomes in *T. gondii* tachyzoites through the recruitment of autophagosomal marker TgAtg8, indicating that autophagy is operating and functional in this parasite.

### Conjugation of TgAtg8 to the autophagosomes is regulated

The conjugation process of Atg8 to the autophagosomal membrane is fairly well understood at the molecular level in yeast and mammalian cells and resembles ubiquitination [8]. In order to bind the autophagosomal membrane, Atg8 must first undergo proteolytic maturation by the cysteine peptidase Atg4 [28], [29]. This exposes a C-terminal glycine that is then conjugated to a phosphatidylethanolamine (PE) lipid moiety through the action of the Atg7–Atg3 system. Accordingly, most eukaryotic Atg8 orthologues described so far bear one or several amino acids after the C-terminal glycine. However, to our surprise, translation of the putative TgAtg8 gene as annotated by the ToxoDB genome database results in a protein that ends in a glycine in the absence of post-translational maturation. This feature was conserved in other predicted apicomplexan Atg8 proteins, with the exception of several *Cryptosporidium* species (Figure S3). To confirm this, we performed a 3′-RACE experiment using *T. gondii* cDNA as template and primers specific for *Atg8* to map the stop codon. In all the clones that we obtained and sequenced, the penultimate codon was confirmed to be coding for a glycine (data not shown). It is conceivable that this peculiar feature may result in constitutive lipidation of Atg8 in *T. gondii* and that maturation by the Atg4 peptidase may not be required. However, analysis of the *T. gondii* genome reveals the presence of a protein with some similarity to Atg4 from yeast (Table S1), and a conserved active site catalytic triad appears to be present. This peptidase, which appears to be expressed in *T. gondii* tachyzoites according to the proteomic data available in the ToxoDB database, could be involved in TgAtg8 recycling from the autophagosomal membrane, but this remains to be investigated.

**Figure 4:**
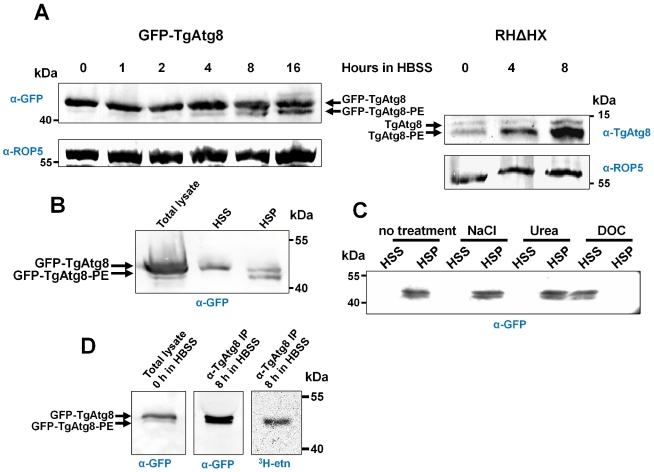
TgAtg8 exists both in soluble and membrane-associated forms that can be separated by urea SDS-PAGE. A. Protein extracts corresponding to tachyzoites incubated in the same starvation conditions as in Figure 2B were separated by urea SDS-PAGE and analysed with an anti-GFP antibody to detect TgAtg8 and the lipidated TgAtg8-PE form. GFP-TgAtg8 parasites extracts were analysed with anti GFP antibody (left), while parental cell line was analysed with anti-TgAtg8 antibody (right). Anti-ROP5 was used as a loading control. B. Cell lysates from GFP-TgAtg8 parasites were subjected to a centrifugation at 100,000 g to separate the soluble fraction (high speed supernatant, HSS) from the membrane fraction (high speed pellet, HSP). The faster migrating form of GFP-TgAtg8 is exclusively present in the membrane fraction as revealed by Western blot analysis after urea SDS-PAGE using anti-GFP. C. The HSP fraction described in B. was extracted with various agents and new HSS and HSP fractions were separated and analysed by Western blot after urea SDS-PAGE. Only a treatment with a detergent (DOC) resulted in GFP-TgAtg8 solubilisation from the HSP fraction. D. GFP-TgAtg8-expressing parasites were grown in host cells in the presence of ^3^H-ethanolamine and then starved in HBSS for 8 hours, still in the presence of the radioactive PE precursor. Tachyzoites were then lysed and GFP-TgAtg8 was immunoprecipitated using anti-TgAtg8 antibody and analysed by urea SDS-PAGE. The immunoprecipitated forms of GFP-TgAtg8 were detected by Western blot using anti-GFP antibody and radioactive ethanolamine was found to be incorporated into immunoprecipitated GFP-TgAtg8, as detected by fluorography (^3^H–etn).

Atg8 and its lipidated form can be separated by SDS-PAGE in the presence of urea [25]. We thus used this technique to separate proteins from GFP-TgAtg8 lysates, followed by Western blot analysis with an anti-GFP antibody. In these conditions, the anti-GFP antibody revealed a major protein band with a molecular size consistent with the prediction for a GFP-TgAtg8 fusion protein (42 kDa) and an additional faster migrating protein (Figure 4A). We used the anti-Atg8 antibody on the parental cell line: we were also able to separate two isoforms of the native protein by urea SDS-PAGE at around 14 kDa (Figure 4A). Lipidated Atg8 typically migrates more rapidly than the non-lipidated form [29], thus the faster migrating protein possibly corresponds to the GFP-TgAtg8-PE form. To confirm that the faster migrating protein was indeed a membrane-associated form of GFP-TgAtg8, we performed cell fractionation experiments that showed the faster migrating form was exclusively present in the 100 000 g insoluble pellet (in contrast, the slower migrating protein was present in both fractions, but appeared to be enriched in the soluble fraction) (Figure 4B). Similar results were obtained on parental cells with the anti-TgAtg8 antibody (Figure S4). Moreover, only a treatment by a detergent (DOC), but not by chemicals disrupting low energy bonds (NaCl and urea), could lead to the complete solubilisation of TgAtg8 proteins present in the membrane fraction (Figure 4C). This has been described for other Atg8s [30] and suggests a tight association of TgAtg8 to membranes. Metabolic labelling using ^3^H-ethanolamine (as a precursor for PE) and subsequent immunoprecipitation of TgAtg8, revealed that TgAtg8 indeed incorporated the label, thus confirming that the membrane association was likely to be mediated by PE (Figure 4D). Also, the abundance of this membrane-associated form increased with the duration of the starvation period (Figure 4A). This confirmed that in *T. gondii* tachyzoites TgAtg8 becomes increasingly lipidated, and hence, potentially, more is recruited to the autophagosomal membranes during starvation (as more autophagosomes are formed).

It is to note that proportions of soluble versus membrane-associated forms of TgAtg8 are higher in the GFP-TgAtg8-expressing cell line, compared to what is generally observed for TgAtg8 in the parental cell line. One explanation is that while the global pool of GFP-TgAtg8 is higher in the GFP-TgAtg8 parasites, the number of autophagosomal vesicles harbouring the membrane-bound form is limited, leaving a significant part of GFP-TgAtg8 in the cytosol.

**Figure 5:**
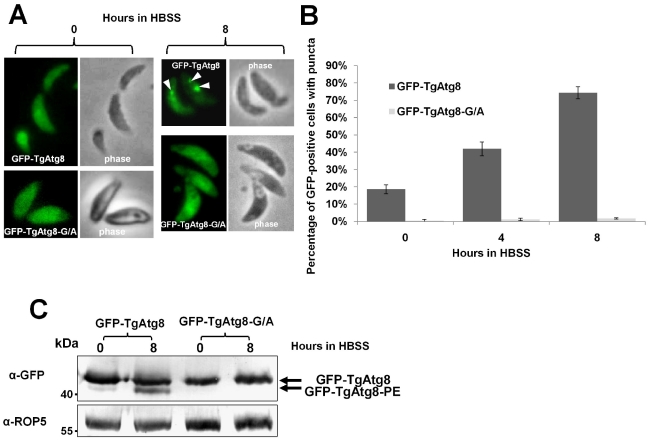
TgAtg8 ends with a C-terminal glycine that is essential for lipidation and conjugation to the autophagosomes. A. Fluorescence microscopy analysis of extracellular tachyzoites expressing GFP-TgAtg8 or its glycine mutant version, before or after induction of autophagy for 8 hours in HBSS. Autophagosomes are labelled by GFP-TgAtg8 in control parasites (arrowheads), but not by the glycine mutant version of the protein. B. Extracellular tachyzoites expressing GFP-TgAtg8 or its glycine mutant version were put to starve in HBSS medium for increased lengths of time and the proportions of cells displaying punctate or cytosolic GFP signals were assessed. Data are mean from *n* = 4 independent experiments ±SEM. C. Protein extracts corresponding to tachyzoites expressing GFP-TgAtg8 and glycine mutant version incubated in HBSS for up to 8 hours, were separated by urea SDS-PAGE and analysed with an anti-GFP antibody to detect GFP-TgAtg8 and lipidated GFP-TgAtg8-PE forms. Anti-ROP5 was used as a loading control.

We then constructed, by site-directed mutagenesis, a variant of GFP-TgAtg8 were we replaced the C-terminal glycine by an alanine. We expected that this should abolish the lipidation of the protein [31]. Tachyzoites were transfected with the GFP-TgAtg8-G/A construct and GFP-positive clones were selected. Autophagy was induced by starvation in amino acids-depleted medium as described above and the appearance of autophagosomes was monitored by fluorescence microscopy (Figure 5A). While the proportion of autophagosome-bearing cells increased along time in the GFP-TgAtg8 control cell line, we did not observe the formation of GFP-labelled autophagosomes in the GFP-TgAtg8-G/A mutant. This finding suggests that the mutated protein is not recruited to autophagosomes (Figure 5A and B), confirming the essential role of the glycine for the recruitment. This finding further validates the interpretation of the GFP-TgAtg8 positive vesicular compartment as autophagosomal. We also followed by Western blot the lipidation of GFP-TgAtg8-G/A following starvation. Consistent with the microscopy results, no lipidated form could be detected for GFP-TgAtg8-G/A by Western blot analysis following urea SDS-PAGE (Figure 5C).

Overall, in spite of a constitutively exposed C-terminal glycine, our results demonstrate that TgAtg8 exists both in soluble and membrane-associated forms in the tachyzoites.

### Autophagy in *T. gondii* tachyzoites during intracellular development

Using GFP-TgAtg8 as a marker we were able to demonstrate that autophagy occurs in extracellular parasites under starvation conditions. However, these are not necessarily reflecting physiological conditions and we wondered whether the parasite might encounter the need for this process during its normal intracellular development. After invasion of the host cell, *T. gondii* tachyzoites replicate inside a parasitophorous vacuole by a process called endodyogeny [32]. This process is composed of single gap phase (G1) preceding a synthesis (S) phase, which is then followed by mitosis and cytokinesis through budding [33]. Daughter cells are assembled within the mother and, as they form, encapsulate most of the maternal cell contents, leaving only a small residual body behind. We hypothesized that some of the maternal material could be digested by autophagy to the benefit of the daughter cells. We thus sought to follow the presence of autophagy in intracellular parasites developing within their host cells in tissue culture. GFP-TgAtg8-expressing tachyzoites were used to synchronously infect host cells [34] and the proportion of intracellular tachyzoites displaying autophagic vesicles was measured as previously at different time points following infection.

**Figure 6:**
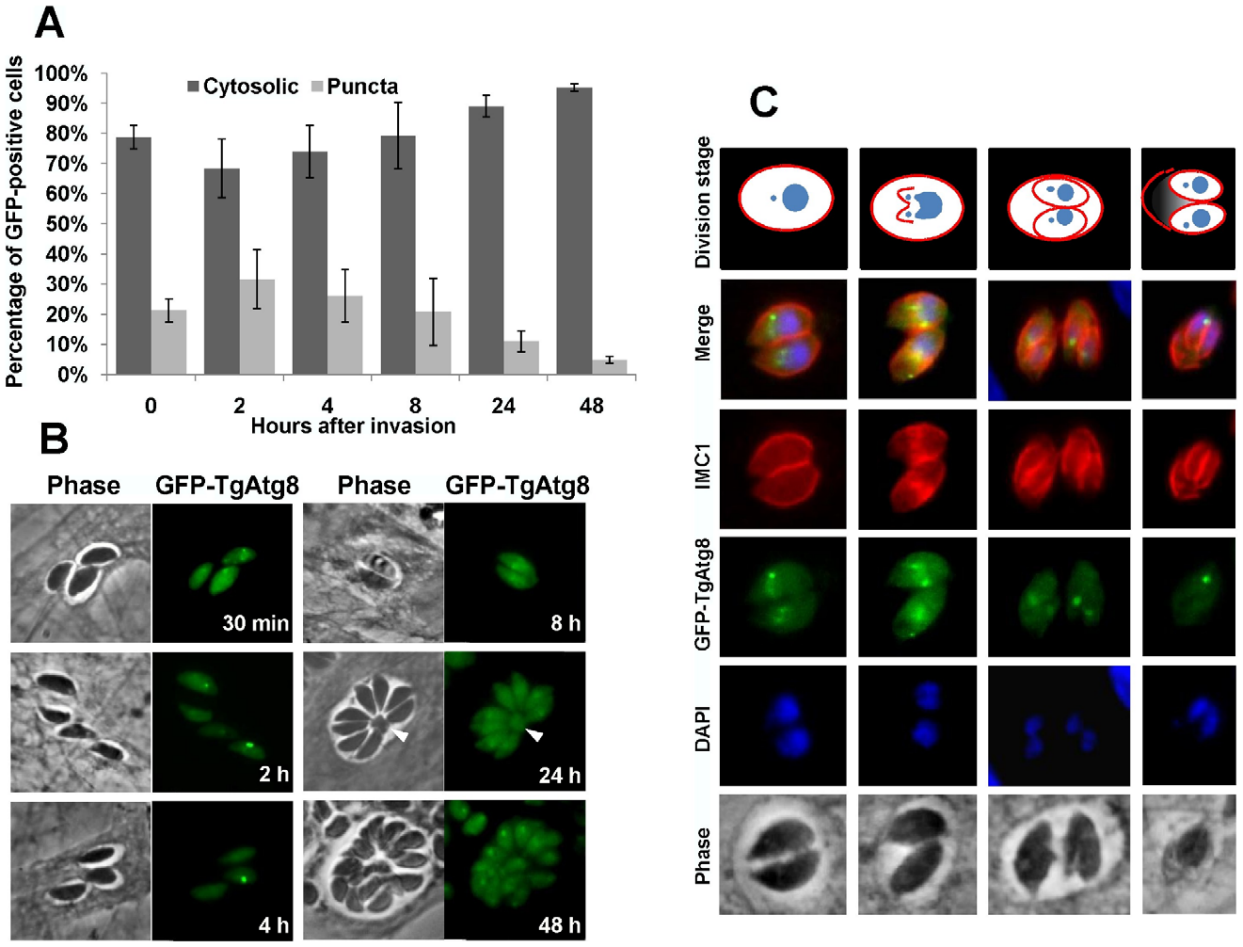
Intracellular tachyzoites display autophagosomes, but these do not seem to be involved in the recycling of mother cell residual material. A. Intracellular tachyzoites expressing GFP-TgAtg8 were used to invade host HFF and the proportions of cells displaying punctate or cytosolic GFP signals were assessed along time during development. Data are mean from *n* = 5 independent experiments ±SEM. B. Fluorescence microscopy pictures of GFP-TgAtg8-expressing tachyzoites during their intracellular development and division. The arrowhead indicates the residual body made of maternal material left after endodyogeny. C. Detection of GFP-TgAtg8-labelled autophagosomes in dividing parasites. Progression of daughter cells division was followed by expression of an IMC1-RFP construct.

It is to note that intracellular parasites were found to display a less intense and a more diffuse GFP-TgAtg8 labelling than the autophagic vesicles induced by starvation, yet slightly more intense than the cytosolic background (Figure 6B, compare 4h and 48 h timepoints). Similar structures were also seen in intracellular parasites expressing non functional GFP-TgAtg8-G/A and were partly co-localising with Golgi apparatus marker GRASP visualised with a red fluorescent protein (RFP) fusion (Figure S5), allowing us to rule out that they were autophagic vesicles. Taking this into account for our quantification experiments, it appeared that there was an initial slight increase in the proportion of cells displaying intensely labelled GFP-TgAtg8 autophagic vesicles, peaking at 2 hours post-invasion. However, such labelling generally decreased in following hours of intracellular development (Figure 6A), where parasites could still be found to display one or several GFP-TgAtg8 autophagic vesicles, but the majority showed a rather homogenous cytosolic signal (Figure 6B). Also, the GFP-TgAtg8 vesicular signal was usually not found at the residual body occasionally formed within the vacuole after parasite division (only in ∼2.5% of the residual bodies) (Figure 6B, arrowed).

To better follow the timing and localisation of the GFP-TgAtg8 signal, we used the inner membrane complex protein IMC1 [35] as a marker to track the progress of cytokinesis. This protein is a component of the subpellicular network that defines the periphery of both the mature tachyzoite and of the daughter cells developing with the mother cell. We imaged GFP-TgAtg8/IMC1-RFP co-expressing tachyzoites, and observed that the vesicular GFP-TgAtg8 signal was usually present in dividing cells (Figure 6C). More precisely, live imaging observation of these parasites showed that autophagosomes were usually present until the cytokinesis process, but disappeared shortly after (data not shown).

Altogether, our data argue against a continuous autophagic activity in intracellular parasites, yet vesicle formations could occasionally be observed and transient autophagy seemed to occur.

### Regulation of autophagy by kinase effectors

Autophagy is a multistep process that can be modulated by upstream kinase effectors and so can be interfered with using specific pharmacological agents. The “target of rapamycin” (TOR) kinase is a serine/threonine kinase which is central in regulating cellular growth by promoting anabolic processes and antagonising autophagy. Consequently, treatment with TOR inhibitor rapamycin mimics nutrient starvation and is known to increase the level of autophagy in several eukaryotes [36]. On the other hand, the class III phosphoinositide 3-kinase (PI3K) signalling cascade has been shown to promote autophagy, thus specifically inhibiting this kinase is thought to reduce autophagy [37]. Both a TOR kinase and a class III PI3K are predicted to be present in the *T. gondii* genome (Table S1); we have thus sought to use specific kinase inhibitors to evaluate the biological significance of autophagy in *Toxoplasma*. We drug-treated GFP-TgAtg8-expressing extracellular parasites and evaluated the effect of treatment on autophagy.

Increasing concentrations of rapamycin in the starvation medium resulted in higher proportions of GFP-TgAtg8 vesicles in extracellular parasites, suggesting an increased level of autophagic activity (Figure S6A). However, the effective concentration was significantly higher than routinely observed in yeast or mammalian cells (5 µg/ml instead of 0.5 µg/ml). This suggests a modest effect of rapamycin on the modulation of *T. gondii* autophagy, like previously observed with the TOR kinase of plants such as rice, tobacco or *Arabidopsis* [38] and the parasitic protist *Trypanosoma brucei* [23].

We also evaluated the effects of class III PI3K on autophagy by incubating extracellular tachyzoites in the starvation solution in the presence of 3-methyladenine (3-MA) or wortmannin, two known PI3K inhibitors. In these conditions, there were lesser proportions of GFP-TgAtg8 vesicles-positive cells amongst the tachyzoites treated with the two inhibitors (particularly wortmannin), which suggested a role for the PI3K in promoting autophagy in *Toxoplasma* (Figure S6B). However, again the concentrations of wortmannin and 3-MA we had to use were higher than those used for mammalian and yeast cells, similarly to plants were these PI3K inhibitors have to be used at greater concentrations to inhibit autophagy [39], [40].

In conclusion, the use of inhibitors specific for kinases known to regulate autophagy in other eukaryotic systems allowed us to show that they generally follow the same trends in *T. gondii* (inhibition of autophagy by the TOR kinase and activation by the class III PI3K), although the use of these drugs at relatively high concentrations can alter their specificity and would preclude a use on intracellular parasites to investigate the autophagic function.

### Isolation of a conditional null mutant for autophagy protein TgAtg3

As we could not use kinase inhibitors to satisfyingly investigate the function of autophagy in *Toxoplasma* tachyzoites, we sought to genetically produce an autophagy-deficient cell line. To this end, we chose *Atg3* as a target, a gene coding for a protein involved in the conjugation of Atg8 and essential for autophagy [41]. In other eukaryotes, Atg8 is activated by Atg7 to form an Atg8/Atg7 thioester intermediate and is then transferred to Atg3 to form an Atg8/Atg3 thioester intermediate, before being finally conjugated to the amino group of PE for binding to the autophagosome membrane.

*TgAtg3* (TGME49_036110, Table S1) was identified in the *T. gondii* genomic sequence based on protein sequence homology searches with eukaryotic orthologues and was found to have a conserved predicted active site region with, in particular, a conserved active site cysteine (Figure S7). *T. gondii* genomic database predicts a 397 amino acids-long TgAtg3 protein. Yet, when aligned to yeast or human orthologues, predicted TgAtg3 shows a N-terminal extension bearing a poly-serine motif (Figure S7). The corresponding putative mRNA region has a polypyrimidine tract (promotes the assembly of the spliceosome), with a putative downstream AG splice acceptor site and a potential downstream start codon that would produce a shorter version of TgAtg3, closer in size to other eukaryotic orthologues.

The regions corresponding to the 3′ and 5′ untranslated regions (UTRs) of *T. gondii TgAtg3* gene were cloned in a plasmid on either side of a selection marker gene to use for a knock-out strategy by gene replacement through a double recombination event. We tried to transfect the RHΔHX strain [42] and the RH-derived ΔKu80 strain, which is more amenable to targeted gene deletion [43]. Despite numerous independent attempts, we were unable to obtain clones in which *TgAtg3* had been deleted (data not shown).

The inability to delete the *TgAtg3* gene with these different strategies suggested an essential role for the corresponding protein in tachyzoites. We thus sought to produce a conditional null mutant cell line for *TgAtg3*. We introduced an ectopic copy of *TgAtg3* into the TAti tet-transactivator line by stable transformation [44]. The ectopic copy was placed under the control of the tetracycline-regulatable promoter 7tetOSag1. To aid detection of the corresponding protein, the transgene was tagged with a sequence encoding an N-terminal c-myc epitope. We cloned both a long and short versions of putative *TgAtg3* into the expression vector (named imyc-lTgAtg3 and imyc-sTgAtg3, respectively). After transfection of tachyzoites of the TAti cell line, clonal parasites were obtained. Both cell lines showed an anhydrotetracycline (ATc)-regulatable TgAtg3 expression by Western blot and by IFA with anti-myc antibody (data not shown). IFA also demonstrated a cytosolic localisation for myc-tagged TgAtg3 (long and short versions alike), which is compatible with a functional role in autophagy, where it should be conjugating cytosolic Atg8 to the autophagosomal membrane (data not shown).

**Figure 7:**
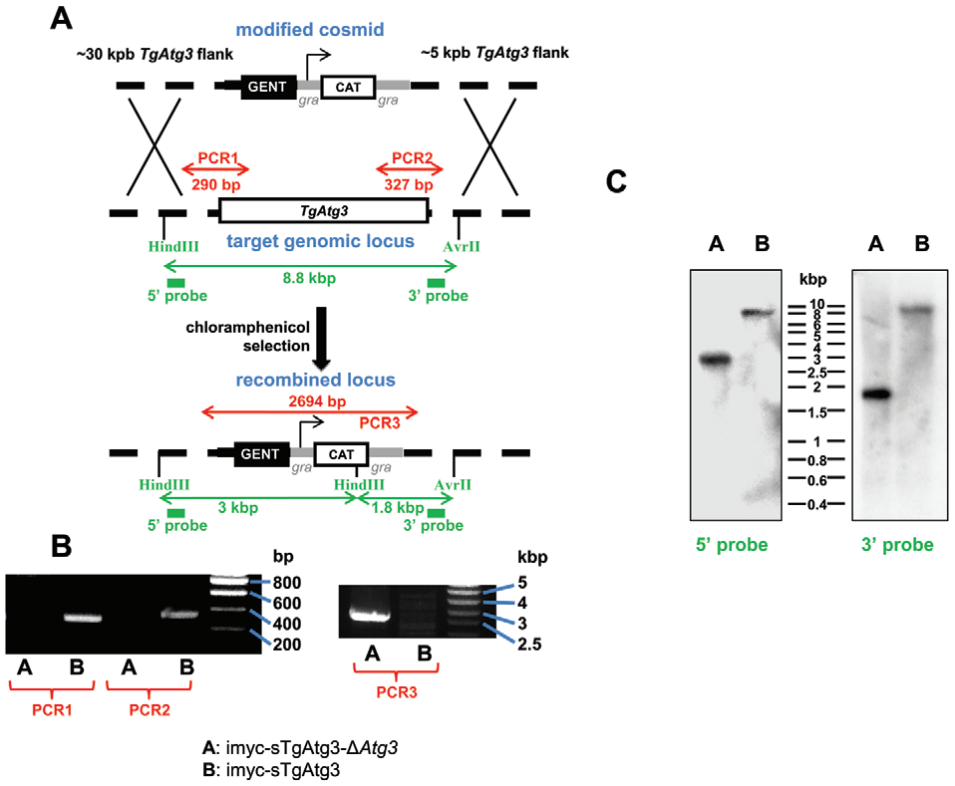
Generation of a *TgAtg3* conditional knock out mutant. A. Schematic representation of the strategy. Recombineered cosmid bearing chloramphenicol acetyl transferase (CAT) gene for selection of resistant parasites, flanked by homologous regions from *TgAtg3*, was used to replace endogenous genomic locus in the cell line expressing a regulatable extra copy of *TgAtg3* (imyc-sTgAtg3). Mutants obtained after double homologous recombination events were selected with chloramphenicol. PCR amplifications and Southern blot strategy used for subsequent characterisation of the mutant are indicated in red and green, respectively. B. PCR verification of the removal of TgAtg3 in the mutant (imyc-sTgAtg3-Δ*Atg3*, A) compared to the parental cell line (imyc-sTgAtg3, B). C. Southern blot verification of the *TgAtg3* gene replacement. ∼4 µg of genomic DNA from mutant (A) and parental (B) tachyzoites were digested by HindIII/AvrII, transferred on a nylon membrane and probed with ^32^P-labeled 5′ and 3′ DNA probes located as schematised in A.

**Figure 8:**
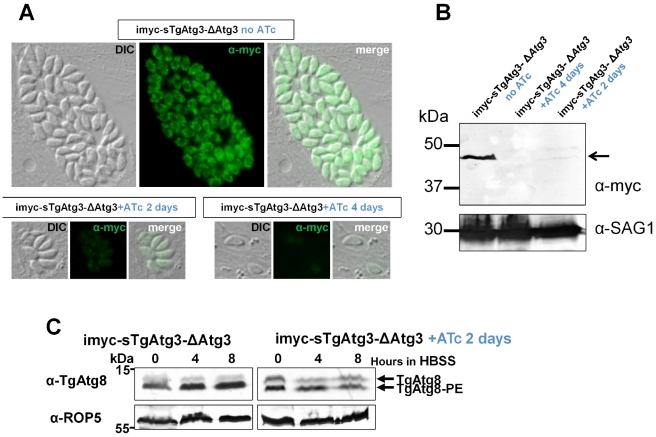
Conditional depletion of TgAtg3 leads to a lack of recruitment of TgAtg8 to the autophagosomes. A. The vector for the expression of the ATc-regulatable sTgAtg3 extra copy allows myc-epitope tagging of this protein. Anti-myc detection of myc-tagged TgAtg3 protein in intracellular parasites either without treatment with ATc, or with a continuous 2 days treatment with 1.5 µg/ml ATc, or after reinvasion following a continuous 4 days treatment. DIC: differential interference contrast. B. Western blot detection of the myc-tagged sTgAtg3 protein (arrowed) in parasite lysates corresponding to the induction conditions described in A. Anti-SAG1 antibody was used as a loading control. C. Western blot analysis following urea SDS-PAGE of TgAtg3-depleted parasites extracts, showing an absence of upregulation of the autophasome-bound TgAtg8-PE form. Anti-ROP5 was used as a loading control.

To replace *TgAtg3*, we used a cosmid-based gene disruption strategy, allowing the use of large flanking regions to increase the chance to recombine at the appropriate locus [45]. A single cosmid clone was available for the *TgAtg3* locus (TOXOU62). In this cosmid the *TgAtg3* open reading frame was replaced with a chloramphenicol acetyl transferase marker by recombineering (Figure 7A). Parasites expressing a regulatable copy of TgAtg3 were transfected with the resulting cosmid and stable clones were isolated by chloramphenicol selection. Clones were tested by PCR for 5′ and 3′ disruption of the *TgAtg3* locus, and presence of the resistance cassette (Figure 7B). We note that we successfully obtained a null mutant clone in the imyc-sTgAtg3 background, but not in the cell line expressing the longer version of TgAtg3. This suggests that this short transcript likely encodes the functional protein. Further analysis by Southern blot confirmed the correct integration of the cosmid-derived cassette and the disruption of native *TgAtg3* locus (Figure 7C). Conditional mutant clone displayed a tight regulation of TgAtg3 expression by ATc, as shown by Western blot and IFA with anti-myc antibody (Figure 8A and B): Western blot analysis showed that a 2 days treatment with ATc was reducing almost completely the expression of the protein and that a 4 days treatment led to undetectable levels of the extra copy.

### TgAtg3 is involved in the conjugation of TgAtg8

We next assessed whether the Atg8-conjugation function of Atg3 was conserved in *T. gondii*. To do so, we used the conditional mutant to deplete intracellular tachyzoites of TgAtg3 by a 2 days treatment with ATc, we then isolated extracellular parasites and triggered autophagy by exposing them to starvation medium for increasing intervals of time as described before. Parasites extracts were prepared and analysed by urea SDS-PAGE to visualise the lipid-conjugated form of TgAtg8. As described above, in the presence of TgAtg3 we observed an increase in the TgAtg8 lipidated form, as autophagy was induced (Figure 8C). In contrast, when TgAtg3 was repressed by ATc treatment: i) a significant proportion of the slower migrating form was present even before autophagy was induced and ii) the lipidated form was not upregulated by starvation (Figure 8C). This shows that reducing the levels of TgAtg3 leads to a reduced capacity in TgAtg8-conjugation to the autophagosomes and thus likely impairs the putative autophagic function in the parasite.

### TgAtg3 is essential for maintaining mitochondrial homeostasis and for growth of *T. gondii* tachyzoites

**Figure 9:**
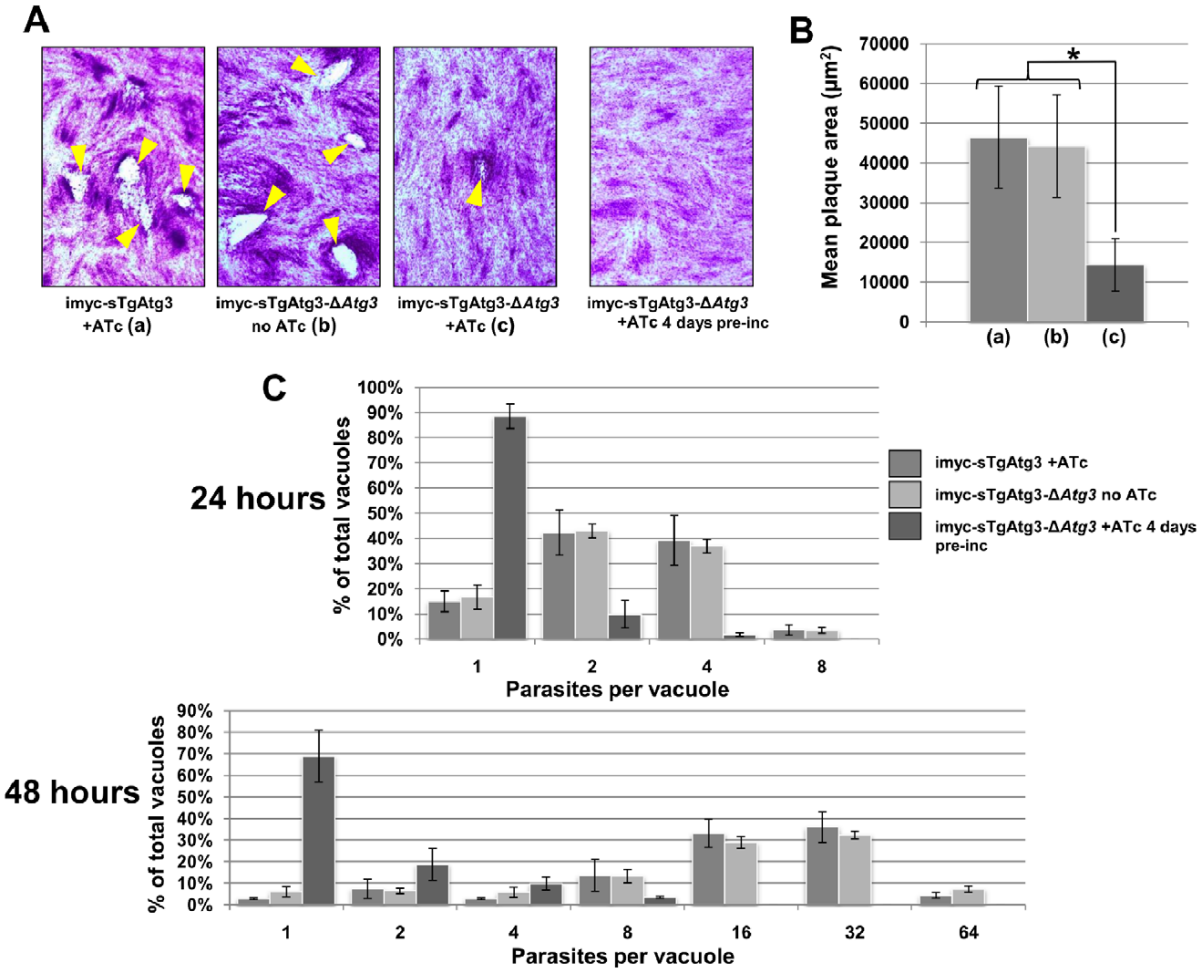
Depletion of TgAtg3 causes a growth defect for intracellular tachyzoites. A. Confluent monolayers of fibroblasts were infected either with extra copy-expressing imyc-sTgAtg3 parental cell line in the presence of ATc (a), imyc-sTgAtg3-Δ*Atg3* mutant cell line without ATc (b), mutant cell line in the presence of ATc (c), or mutant cell line pre-incubated with ATc before start of the experiment and maintained in the presence of ATc. Plaques (arrowheads) resulting from the lysis of host cells due to the multiplication of the parasites are only visible when TgAtg3 is still expressed. B. Mean plaque area comparisons between fibroblast layers infected with controls and TgAtg3-depleted parasites (a, b, c: legend as in A). Plaques observed with the mutant in the presence of ATc were significantly smaller than with control cell lines (* p<0.005, Student's T test). Data are mean from *n* = 3 independent experiments ±SEM. C. Numbers of parasites per vacuole are significantly lower in TgAtg3-depleted cell line (pre-incubated for 4 days with ATc) compared with controls 24 or 48 hours post invasion. Data are mean from *n* = 3 independent experiments ±SEM.

We examined the effect of TgAtg3 depletion on parasite growth using plaque assays. The conditional TgAtg3 null mutant formed markedly smaller plaques when repression of the imyc-sTgAtg3 copy was induced by ATc (Figure 9A and B). Longer repression periods increased the effect on growth, as no plaque was visible with mutant parasites pre-incubated with ATc for 4 days prior to the start of the plaque assay (Figure 9A). No viable parasite could be detected after three passages following mechanical release, in the presence of ATc (data not shown), providing additional support for a critical function of TgAtg3. Growth was also assessed at shorter times following invasion. Mutant parasites that were untreated or pre-incubated with ATc for 4 days were allowed to invade host cells. Cultures were kept in the presence of ATc and fixed 24 or 48 hours later and numbers of parasites per vacuole were counted in both samples. TgAtg3-depleted parasites showed a considerable delay in growth compared to controls, and they did not progress through cell division as they accumulated vacuoles with mostly one or two parasites (Figure 9C).

**Figure 10:**
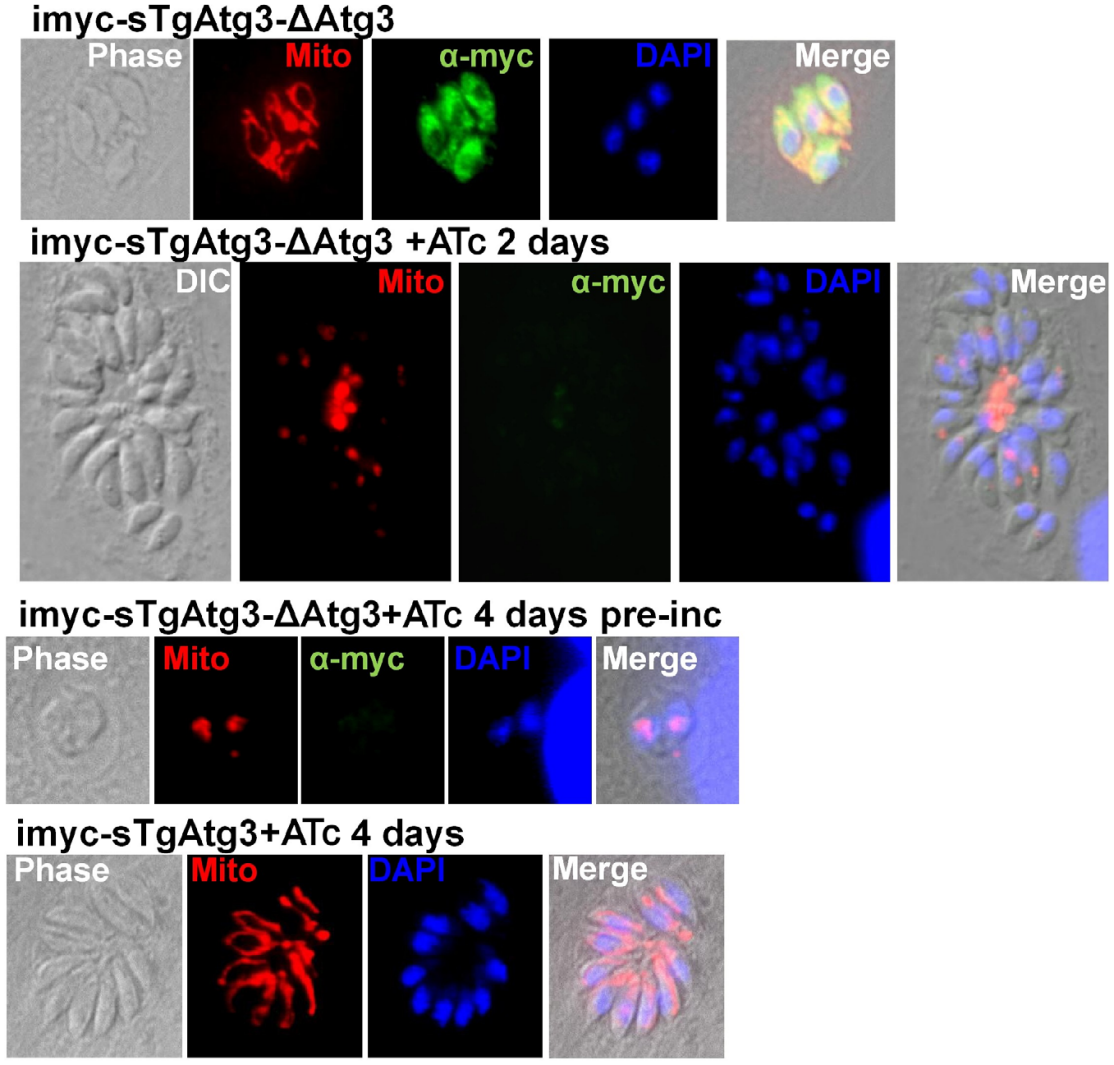
TgAtg3-depleted parasites show a defect in mitochondrion morphology. Mitochondrion labelled with specific antibodies (see results section) was found as a reduced structure, or even absent, in TgAtg3-depleted cell lines (middle series of micrographs, kept for 2 days in the presence of ATc or pre-incubated with ATc for 4 days before invasion, respectively). Parasites left to develop for two days in the presence of ATc to progressively extinguish the expression of TgAtg3 showed an accumulation of mitochondrial marker at the residual body. TgAtg3 depletion was verified by detecting myc-tagged regulatable extra-copy with specific antibody. TgAtg3-expressing cell line imyc-sTgAtg3cultivated in the presence of ATc for 4 days was used as a control for mitochondrial morphology (bottom). DNA was labelled with DAPI.

As the intracellular development of tachyzoites appeared to be affected by TgAtg3 depletion, we sought to investigate whether this was associated with specific morphological defects. We performed IFA using a battery of antibodies recognising specific subcellular structures. These included secretory organelles such as the rhoptries, micronemes and dense granules, the IMC, the apicoplast and the mitochondrion (Figure S8 and data not shown). The organelle that stood out in these comprehensive analyses as being particularly affected by the lack of TgAtg3 was the mitochondrion. *T. gondii* tachyzoites typically have a single mitochondrion, which forms a reticulated network extending through most of the parasite (Figure 10, top, bottom). Strikingly upon depletion of TgAtg3, we observed the loss of the mitochondrial network as judged by staining for two independent mitochondrial marker proteins: F1 beta ATPase and HSP28 (Figure 10). In parasites that were grown for 2 days in the presence of ATc, the mitochondrion appeared highly fragmented or entirely absent. We note that a significant amount of staining was now found in the residual body. This structure is, located at the center of the parasitophorous vacuole and is thought to represent the residua of mother cells left behind by the emerging daughters (Figure 10). We confirmed this phenotype in transgenic parasites pre-incubated for longer periods with ATc, but it was not generally seen in TgAtg3-expressing control parasites exposed to ATc (Figure 10). To independently assess the morphology and the functional status of the mitochondrion in this mutant we performed labelling experiments with Mitotracker Red. This cationic dye accumulates in the mitochondrion depending on an intact membrane potential. We noted a profound loss of staining consistent with a concomitant loss of mitochondrial membrane potential and/or loss of the organelle (Figure S9).

No obvious defect could be detected for the apicoplast or the secretory organelles (Figure S8). We therefore assume that this phenotype reflects a specific role of Atg3 in mitochondrial maintenance rather than a general loss of cell structure due to necrosis and cell death. However, more subtle effects might be beyond the limit of resolution of fluorescence microscopy. Therefore we sought to analyse the morphology of TgAtg3-depleted parasites by electron microscopy.

**Figure 11:**
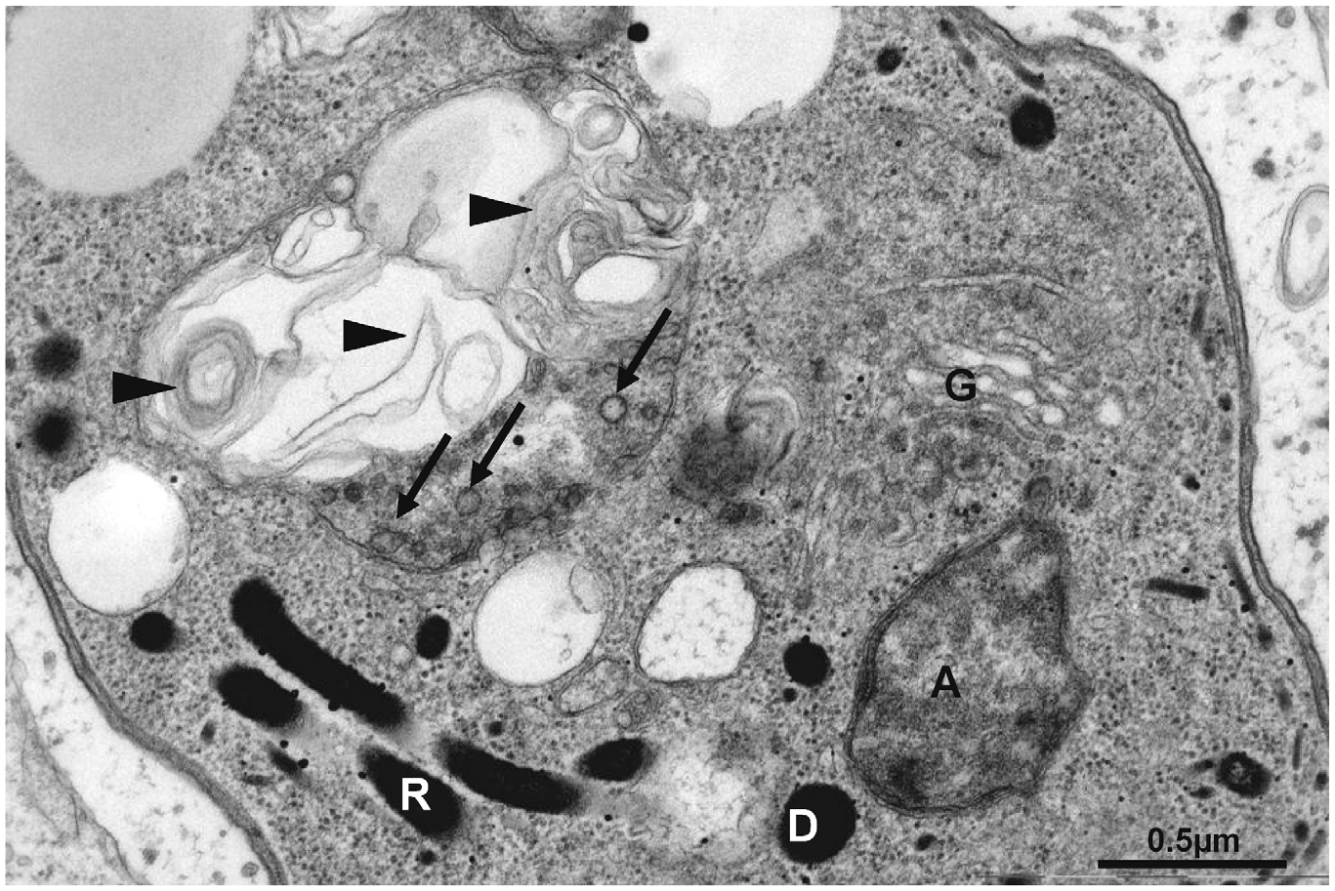
Altered mitochondrial ultrastructure in TgAtg3-depleted tachyzoites observed by electron microscopy. Ultrathin section of a TgAtg3-depleted (3 days of ATc treatment) tachyzoite showing the dramatic alteration of the mitochondrion, where cristae remnants are still visible (arrows), but which is filled up with membranous profiles (arrowheads), whereas Golgi (G), apicoplast (A), rhoptries (R) and dense granule (D) look normal.

Electron microscopy confirmed that these tachyzoites possessed morphologically normal secretory organelles (rhoptries, micronemes and dense granules) and apicoplast. We examined parasites grown in the presence of ATc for up to 4 days and found no apparent detriment to these organelles over this period (Figure 11 and data not shown). In contrast, electron microscopy revealed numerous mitochondrial defects ranging from alteration of cristae, to organelles including large collapsed membranous structures next to vestigial cristae that were the only recognisable feature of the mitochondrion (Figure 11). Altered mitochondrial material was also occasionally present in the residua found in the vacuolar space (not shown).

In conclusion, loss of TgAtg3 impedes the intracellular development of parasites and the most visible effect at the subcellular level is a dramatic collapse of the parasite mitochondrion.

## Discussion

### The molecular machinery of autophagy in *T. gondii*

Autophagy plays a role in organelle and protein turnover that is important for cellular homeostasis, adaptation to starvation and overall cellular development of eukaryotic cells. Yet, it was to date poorly characterised in apicomplexan parasites. Autophagy is dependent on the formation of the autophagosome, the vesicular structure in which the material to be degraded will be incorporated. In the present study we have used a molecular marker for autophagosomes, TgAtg8, to detect these structures in *T. gondii*. We have identified structures that show the morphological hallmarks of autophagosomes and can be formed and induced by starving extracellular tachyzoites; more importantly, we find that autophagosomes are also formed during normal development and that their presence is most pronounced at a particular point in the parasite division cycle.

Our genome mining efforts to uncover autophagy-related genes in *T. gondii* and other Apicomplexa have revealed a core of the autophagosome formation machinery in these parasites. We identified genes coding for proteins involved in the pre-autophagosome formation, the Atg8 conjugation system for autophagosome elongation, as well as upstream regulating kinases. However, several proteins previously described to regulate the formation of autophagosomes in other eukaryotic systems appear to be missing. Examples of these are the Atg5–Atg12 conjugation system for Atg8, or partners of TOR-regulated Atg1 kinase (i.e. Atg13, Atg17). This suggests that Apicomplexa possess a simpler system for autophagy that lacks some of the complex layers of regulations observed in mammalian cells or yeast [46] or, alternatively, that the regulation of autophagy in Apicomplexa involves parasite-specific proteins that remain to be discovered. Along this line, our use of inhibitors to interfere in *T. gondii* with known upstream autophagy-regulating kinases (inhibiting mTOR kinase and activating class III PI3K) has shown that, although following the same trends as their other eukaryotic counterparts, high concentrations of inhibitors were needed to act upon autophagy, suggesting differences in the kinase-dependent regulatory network. The structure of the Atg8 protein in *T. gondii* and many other Apicomplexa may be consistent with this apparent differential regulation. In yeast and mammals, Atg8 first needs to be processed by the Atg4 cysteine peptidase in order to bind to the nascent autophagosome membrane. This exposes a C-terminal glycine residue that will, in turn, be conjugated to a lipid by the Atg7–Atg3 complex. Yet, most apicomplexan Atg8 orthologues already constitutively bear a C-terminal glycine, which could suggest that autophagosome formation is constitutive in Apicomplexa. However, our experiments suggest some level of regulation of TgAtg8 binding to the autophagosomes, and this could occur by a mechanism independent of C-terminal proteolysis. Firstly, using urea PAGE, we could separate the non-lipidated from the lipidated isoform of GFP-TgAtg8 and native TgAtg8, and further showed that the proportions of the membrane-bound form were increasing in autophagy-inducing conditions. Secondly, microscopic observation of GFP-fused or native TgAtg8 showed dual vesicular and cytosolic localisations of the protein. Lastly, using ectopic expression of GFP-TgAtg8 mutants, we demonstrated that the C-terminal glycine was essential for conjugation.

Overall, our data suggest that autophagy in *T. gondii* is more complex than it appears at first glance and could have original ways of regulation compared with other eukaryotic systems studied so far.

### What is the role of autophagy in *T. gondii*?

As they invade their host cell, *T. gondii* tachyzoites establish themselves into a parasitophorous vacuole, which constitutes a niche that offers protection and nutrients and thus allows for efficient parasite multiplication (see [47] for a review). Spatial reorganisation of host organelles and cytoskeleton around the parasitophorous vacuole is observed within minutes following entry and it almost certainly plays a role in parasite nutrient acquisition. It is thus unlikely that the parasite, once intracellular, is experiencing starvation. We have produced a TgAtg3 conditional mutant that appears unable to regulate the conjugation of TgAtg8 and thus is likely deficient in autophagy. TgAtg3-depleted parasites invade host cells, and divide once or twice. Therefore TgAtg3-dependent autophagy does not seem to be essential in early phase of infection; however parasites cease to grow rapidly, indicating an important role for continued intracellular development. As for extracellular tachyzoites, once they have egressed their host cell, their fate is highly dependent on their motility and their ability to quickly invade a neighbouring cell or tissue, and although little is known about the exact environmental conditions encountered within its vertebrate host, autophagy could also be promoting the ability of *T. gondii* to survive during the journey of the parasite within the host tissues and organs.

Autophagy can occur in response to cellular stresses such as starvation, but there is growing evidence for more specific needs for autophagy to maintain cell homeostasis during normal growth and development. This can be directed towards specific organelles such as the mitochondrion (mitophagy) or the peroxisomes (pexophagy) [48]. This type of selective autophagy is used by cells to dispose of damaged organelles, or to clear the cell from these organelles when undergoing differentiation. In that context, we thought that there could be a targeted role for autophagy in the intracellular development of *Toxoplasma*. Some of the organelles of the mother cell are made redundant by their *de novo* formation in the daughters (i.e. rhoptries, micronemes) and we hypothesized that these might be degraded and recycled through autophagy. Interestingly, the use of PI3K inhibitor 3-MA on intracellular tachyzoites has been recently shown to affect parasite division, particularly daughter bud formation [49]. Also, morphological observations of 3-MA-treated parasites showed considerable retention of cellular material in residual body-like structures during daughter cell formation. It is to note that the authors showed that wortmannin did not produce similar effects, although in our hands wortmannin was a more potent inhibitor of autophagy than 3-MA on extracellular parasites, however wortmannin is notoriously unstable in culture over long periods of time and could have been degraded in the 20 hours long incubation used in their protocol, thus preventing lasting effects [50].

When depleting the parasites of TgAtg3, no accumulation of organelles or morphological alteration of *de novo* synthesized organelles was observed, suggesting that TgAtg3-dependent autophagy is not required for the recycling of secretory organelles from the mother. Nevertheless, TgAtg3 depletion has a significant cellular phenotype: the structural and functional alteration of the single mitochondrion present in the tachyzoites.

Is the break up of the mitochondrion that we observe in this mutant a cause or a consequence of the growth arrest of the tachyzoites? Although mitochondria are generally considered the powerhouse of the cell, many intracellular parasites rely heavily on glycolysis for energy production and do not require oxidative phosphorylation. The relative importance of the mitochondrion for energy generation in Apicomplexa is disputed, although the presence of a mitochondrial membrane potential (detected with Mitotracker labelling for instance) suggests it is at least partly functioning. However the other, often overlooked, metabolisms possibly hosted by the mitochondrion or in close interaction with the neighbouring apicoplast (i.e. redox metabolism, heme synthesis) are essential for the survival of the tachyzoites. On the other hand, the mitochondrion plays an active role in mediating apoptosis-like cell death, even in unicellular eukaryotes such as yeast [51], but this area remains largely unexplored in *Toxoplasma*. When treating intracellular tachyzoites for up to two days with dihydrofolate reductase inhibitor pyrimethamine [52], we could observe that cells having already lost their overall morphology, their normal micronemal distribution and displaying a fragmented nuclear content, still retained a reticulated mitochondrial signal (Figure S10), indicating that mitochondrial fragmentation is not necessarily an early event in the course of tachyzoite cell death. Moreover, when TgAtg3-depleted parasites (i.e. after 4 days of ATc treatment) were left to invade hot cells, parasites with a fragmented mitochondrion were found intracellularly after short invasion times (15 minutes), demonstrating that, in spite of the perturbation of mitochondrial homeostasis, these tachyzoites had retained their invasive potential (data not shown). Along with this, a recent work has been illustrating that glycolysis, but not mitochondrion-associated oxidative phosphorylation, was the major contributor to ATP production and was necessary to maintain the invasive potential of extracellular tachyzoites in carbon-depleted medium [53].

Mitochondrial dynamics and mitochondria turnover are linked. In yeast, but also mammalian cells, mitochondria undergo fusion/fission events during the cell cycle, for exchange of metabolites or DNA [54]. During these events, “maintenance” mitophagy is used to eliminate damaged mitochondrion and is intimately linked with fission events [55], which lead to perturbation of mitochondrial membrane potential. This way, depolarised mitochondria are eliminated and autophagy contributes to normal mitochondria homeostasis [56]. The replication and dynamics of *T. gondii* mitochondrion have been poorly studied; it is not known how the organelle elongates and to which extend fusion/fission events occur in the parasite. A recent report [57] has shown that the replication of the organelle was tightly coupled with the cell division cycle and that the mitochondrion was elongating after daughter scaffold formation, before entering the daughter cells at a very late stage. The appearance of the autophagosomal structures we have observed in *Toxoplasma* tachyzoites seems to be within the timeframe of mitochondrion replication/division. Consistent with this, recent cell-cycle microarray data [58] searchable through ToxoDB release 6.2 (www.toxodb.org) have revealed that, although TgAtg8 levels seemed to vary little during the cell cycle, autophagosome-conjugating partners TgAtg3 and TgAtg7 had similar profiles and were predominantly expressed towards the end of the G1 phase. This is compatible with an increase in autophagosomal activity after this part of the cell cycle, with a peak during the end of the S phase and the mitosis. Our microscopic observations also suggest the occurrence of mitophagy in the parasites. It thus seems that maintaining mitochondrial homeostasis during the biogenesis of this organelle in tachyzoites necessitates TgAtg3-dependent autophagy, and that loss of this autophagic function could lead to mitochondrion break up, loss of membrane potential and cell death. Interestingly, a recent report [59] has shown that a mammalian *Atg3* null mutant also displays increased levels of fragmented mitochondria, and that Atg3 regulates mitochondrial homeostasis through an association with autophagy protein Atg12 (which appears to be absent from *T. gondii*'s genome). Another recent study has suggested that cell death induced by starvation in autophagy-defective yeast mutants was caused by a dysfunction of the mitochondria [60]; more precisely, it showed that autophagy mutants accumulated high levels of reactive oxygen species and experienced defects in their respiratory functions.

In summary, our study shows that autophagy is present and functioning in the apicomplexan parasite *T. gondii* and that protein TgAtg3, through its role in TgAtg8 conjugation to the autophagosome, is likely essential for autophagy. Moreover, its function is crucial for maintaining mitochondrial homeostasis and for parasite growth. Many questions remain unanswered regarding the physiological roles of autophagy for *T. gondii* and Apicomplexa in general, or the machinery they use for this particular cellular function. For instance, the drastic phenotype we have observed with TgAtg3-depleted parasites is not exclusive and autophagy could still have a role in regulating parasite organelles other than the mitochondrion in specific physiological conditions. It is for instance possible that autophagy plays a role for organelle clearance during conversion from a parasitic stage to another. In related Apicomplexa *Plasmodium*, sporozoite to trophozoite conversion in the liver involves quite an extensive clearance of superfluous organelles and shape remodelling, in which autophagy is possibly playing a role [17]. Infectious stages of *T. gondii*, sporozoites, tachyzoites, and bradyzoites, are rather similar ultrastructurally but differ in certain organelles (for instance tachyzoites have more dense granules than bradyzoites, but have less micronemes and amylopectin granules) as well as their metabolic state and could be dependent on autophagy for differentiation and cell remodelling. Also, the role of autophagy during oocyst sporulation should be investigated. Again, *in vivo* studies using the TgAtg3 conditional mutant we have generated could reveal interesting features.

Also, one important question concerns the degradation and recycling of autophagocytosed material in *Toxoplasma*. Indeed, once completed, the double-membrane autophagosome is transported to a hydrolases-containing compartment and the outer membrane of the vesicle fuses with this compartment for degradation of its content. Typically, this lytic compartment is the lysosome in mammalian cells (or the vacuole in yeast), but so far the presence of such a compartment has remained elusive in *T. gondii* tachyzoites. Recent findings have nonetheless identified a compartment that seems to bear several characteristics of a *bona fide* late endosomal/lysosomal compartment (i.e. acidified, containing a peptidase) [13], [14], however this compartment is changing both in aspect and contents during the tachyzoite cell cycle, which renders it difficult to grasp. Moreover, the interactions between this compartment and autophagosomes are supposedly transient, so the interplay between the two would require dynamic studies on live cells. The repertoire of hydrolases present in the lytic compartment of tachyzoites and the ones particularly involved in the degradation of autophagic material also remain to be characterised.

Our discovery that autophagy protein TgAtg3 is essential for intracellular development of the parasite opens a new area for looking into possible parasitic drug targets, especially given the apparent peculiarities in the regulation of the parasite autophagic machinery compared with its host counterpart and the fact that this machinery contains enzymes (kinases, peptidases) for which inhibitors could be screened.

## Materials and Methods

### Ethics statement

This study was conducted according to European Union guidelines for the handling of laboratory animals and the immunisation protocol for antibody production in rabbits was conducted at the CRBM animal house (Montpellier) and approved by the Committee on the Ethics of Animal Experiments (Languedoc-Roussillon, Montpellier) (Permit Number: D34-172-4, delivered on 20/09/2009).

### Host cells and parasite culture

Tachyzoites of the RHΔHX strain, deleted for hypoxanthine guanine phosphoribosyl transferase [42] or ΔKu80 strain [43] and derived transgenic parasites generated in this study, were propagated *in vitro* under standard procedures by serial passage in human foreskin fibroblasts (HFF) monolayers in Dulbecco's modified Eagle medium (DMEM, with 4500 mg/l D-Glucose, sodium pyruvate, Invitrogen) supplemented with 10% fetal bovine serum and 2 mM L-glutamine.

### Cloning of *T. gondii* GFP-fused Atg8 and generation of mutant version

Total RNA was isolated from *T. gondii* tachyzoites using the RNAqueous kit (Ambion), according to the manufacturer's instructions. cDNA was synthetized from the isolated RNAs by reverse transcription using random hexamers and the SuperScript II kit (Invitrogen) or using the SMART RACE cDNA Amplification Kit (Clontech Laboratories). DNA corresponding to *T. gondii* Atg8 orthologue (TGME49_054120, http://toxodb.org) was obtained by PCR from cDNA with primers ML303/ML304 (see Table S2 for primer sequences) bearing the PstI and PacI restriction sites, respectively. The fragment was cloned into the pTGFP vector [61] to bear the pGFP-TgAtg8 plasmid for expression of Atg8 in *Toxoplasma* with the green fluorescent protein (GFP) fused at its N-terminus.

C-terminal glycine mutant version of the GFP-TgAtg8 construct was obtained by site-directed mutagenesis with the QuikChange mutagenesis kit (Stratagene), with primers ML308/ML309 to yield plasmid pGFP-TgAtg8-G/A with the C-terminal glycine mutated to an alanine. All constructs were checked by sequencing.

### Generation of GFP-TgAtg8-expressing *T. gondii* cell lines

To generate stable transformants, 5×10^7^ extracellular tachyzoites of the of the RHΔHX strain were transfected and selected as previously described [42]. GFP-TgTgAtg8-expressing parasites were obtained by electroporation of 100 µg of plasmids for the expression of GFP-TgTgAtg8 or its mutated version. After overnight growth, transfectants were selected with 25 µg/ml mycophenolic acid and 50 µg/ml xanthine for three passages, before cloning by limiting dilution under drug selection. After expanding the clones, GFP-expressing parasites were selected by observation with a fluorescent microscope.

### Production of an anti-TgAtg8 antibody

A DNA sequence corresponding to the full TgAtg8 protein was obtained by PCR from *T. gondii* RH tachyzoites cDNA with primers ML697 and ML698. It was then cloned into pGEX-4T-3 (GE healthcare) and the construct was transformed into *E. coli* BL21 cells to produce a recombinant protein with an N-terminal glutathione-S transferase tag, which was used to immunise a rabbit. The antibody was subsequently used at 1/500 for Western blot or IFA.

### Molecular cloning and generation of autophagy-deficient mutants

Two approaches were used for knock-out of autophagy-related genes by double homologous recombination events. First, a plasmid bearing 5′ and 3′ untranslated regions (UTR) of the gene of interest flanking a selection cassette was generated. 5′ and 3′ UTR were obtained by PCR. They were cloned on either side of the chloramphenicol acetyl transferase (CAT) gene in the pTub5/CAT vector, serving as a selection marker [62]. Primer pairs used for PCR amplification were ML358/ML355 ML339/ML340 for 5′ and 3′UTR, respectively, of *TgAtg3*.

Second, cosmid with larger flanking regions to increase the frequency of homologous replacement was generated. Cosmid recombineering was performed as described previously [45]. Briefly, a cosmid overlapping *TgAtg3* (TOXOU62) was recombineered with a cassette bearing a selection marker and obtained from plasmid template pH3CG by PCR, with primers ML537/ML538. DNA constructs for gene replacement were transfected into either RHΔHX or ΔKu80 strains and selected with the appropriate antibiotic.

For conditional knock-out strategy, an ectopic copy of *TgAtg3* was introduced under the dependence of a SAG1 promoter. Two alternative start codons (see Figure S5) were tried for the constructs, corresponding to a long (amplified with ML454/ML456) or shorter (ML455/ML456) version of the *TgAtg3*. Constructs were introduced into the TAti tet-transactivator cell line by stable transformation [44]. For repression of the expression of the extra-copy, anhydrotetracycline (Clontech) was put at 1.5 µg/ml in the culture medium for two to four days.

### Verification of *TgAtg3* locus disruption

Correct disruption of the *TgAtg3* locus was verified by PCR using primers ML595/ML654 (PCR1), ML386/ML387 (PCR2), ML595/ML596 (PCR3). Primers ML650/ML651 and ML648/ML649 were used to generate the 5′ and 3′ probes, respectively, used for Southern blot analysis.

### Induction, modulation and monitoring of autophagy

To induce autophagy, extracellular tachyzoites were put in starvation conditions. Extracellular parasites were obtained from freshly lysed HFFs, sedimented by centrifugation and washed twice in Hank's Balanced Salt Solution (HBSS) before being resuspended in pre-warmed HBSS and incubated at 37°C for up to 16 h.

Autophagy was occasionally modulated by incubation of the cells with several effectors: PI3K inhibitors Wortmannin and 3-methyladenine (Sigma) at 10 µM and 10 mM, respectively; TOR kinase inhibitor rapamycin (Santa Cruz), at concentrations up to 5 µg/ml.

Autophagosomes were quantified in live or paraformaldehyde-fixed GFP-TgAtg8 expressing parasites, by microscopic observation and counting of the punctate GFP signals. At least 200 cells were counted in each experimental set. Alternatively, the presence of the lipidated, autophagomal membrane-associated, form of GFP-TgAtg8 was assessed by Western blotting with anti-GFP antibody after separation by urea SDS-polyacrylamide gel electrophoresis (see below). Parasites extracts were normalised on counts of viable parasites (by trypan blue assay) at the end of the incubation time.

### Subcellular fractionation

3.10^7^ GFP-TgAtg8 tachyzoites starved for 8 hours in HBSS were solubilised in 1 ml of Tris HCl 50 mM pH 7.5 and sonicated twice for 30 seconds. Cellular debris were removed by centrifugation at 500 g for 10 minutes. The supernatant was submitted to an ultracentrifugation at 100 000 g for 30 minutes to yield a membrane-enriched high speed pellet and high speed supernatant soluble fractions, respectively. The supernatant fraction was TCA-precipitated and extracts were resuspended in SDS-PAGE loading buffer prior to Western blot analysis.

Alternatively, the high speed pellet was further extracted by 1 M NaCl, 2 M urea or 1% deoxycholate (DOC) for 4 hours at 4°C and submitted to another ultracentrifugation to yield a pellet and supernatant fraction.

### Western blot analysis

Western blots were performed as described previously [63], with the modification that urea was included at a concentration of 6 M in the SDS-polyacrylamide gel to separate lipidated and non-lipidated forms of GFP-TgAtg8. The primary antibodies used for detection and their respective dilutions were: anti-GFP monoclonal mouse antibody (Roche) at 1/500, and anti-ROP5 [64] at 1/1000 as a loading control.

### *In vivo* labelling and immunoprecipitation of TgAtg8

One 75 cm^2^ flask of HFF was grown for 24 hours with 0.1% fetal bovine serum, in the presence of 120 µCi of [1–^3^H] Ethan-1-ol-2-amine hydrochloride (GE Healthcare). HFFs were then infected for 24 hours with 5.10^7^ GFP-TgAtg8 parasites; HFF layer was scrapped and parasites were syringed out. Isolated parasites were washed once in HBSS and incubated for 8 hours in 10 ml of HBSS, still in the presence of 120 µCi of labelled ethanolamine. They were washed once in HBSS and the pellet was resuspended in 1.5 ml of lysis buffer (PBS with 1% Nonidet 40 (NP40), 0.5% DOC and 0.1% SDS with a protease inhibitors cocktail (Roche)) and incubated at 4°C for 1 hour. The lysate was centrifuged for 20 min at 15 000 *g* and the supernatant was collected for subsequent immunoprecipitation.

Protein A-sepharose beads (Sigma) were prepared by putting together 50 µl of polyclonal rabbit anti-TgAtg8 antibody with 20 µl of beads for 1 hour. They were then washed 3 times in PBS to eliminate unbound antibodies.

1.5 ml of radiolabeled lysate was incubated with protein A-bound anti-TgAtg8 antibody overnight at 4°C under gentle agitation, and then washed five times with lysis buffer. Remaining buffer was discarded and the beads were resuspended in 20 µl of SDS-PAGE loading buffer and boiled for 5 minutes before analysis by urea SDS-PAGE. The gel was treated with Amplify (GE healthcare), dried and used for fluorography.

As a control, 5.10^7^ parasites were treated in a similar way except that no radioactive ethanolamine was used during growth and starvation and urea SDS-PAGE, followed by Western blot analysis with anti-GFP antibody, were used after immunoprecipitation.

### Fluorescent staining of cells

IFAs were performed either on extracellular tachyzoites recovered from freshly lysed HFF, or intracellular parasites at their various stages of development. They were fixed in 4% (w/v) paraformaldehyde in PBS and processed for immunofluorescent labelling as described previously [63], with the modification that the extracellular parasites were made to adhere onto poly-L-lysine slides for 20 minutes prior to processing for immunofluorescent labelling.

The following antibodies were used at 1/1000 dilution unless mentioned: anti-mitochondrial F1 beta ATPase (P. Bradley, unpublished), anti-mitochondrial HSP28 [65], anti-acyl carrier protein [66], anti-MIC3 [67], anti-ROP5 [64], anti-c-myc at 1/250 (Santa Cruz).

For co-labelling with fluorescent markers in live cells, constructs allowing the expression of IMC1 fused to the Tomato variant of RFP (B. Striepen, unpublished) and GRASP-RFP [57] were transfected in GFP-TgAtg8-expressing tachyzoites.

Fluorescent labelling of the mitochondrion was performed on extracellular parasites using MitoTracker Red CMXRos (Invitrogen) at 50 nM for 30 minutes at 37°C. Tachyzoites were then washed extensively in HBSS, fixed in 4% (w/v) paraformaldehyde in PBS and adhered onto poly-L-lysine slides before microscopic observation. For labelling of the mitochondrion in intracellular parasites, MitoTracker Red CMXRos was used at 500 nM for 45 minutes at 37°C and chased for 15 minutes 37°C before cells were processed for immunolabelling and microscopic observation.

Slides were mounted with Immumount (Calbiochem) and observed either with a Leica DMRA2 microscope, and images acquired with a MicromaxYHS1300 camera (Princeton Instruments) using the Metamorph software (Molecular Devices) or with an Axiovert/200M Zeiss inverted microscope equipped with an Axiocam MRm CCD camera (Zeiss) driven by the Axiovision software. Image acquisition was performed on workstations of the Montpellier RIO Imaging facility.

### Electron microscopy

Parasite pellets were fixed for 2 hours with 2.5% glutaraldehyde in 0.1 M Na cacodylate buffer pH7.2, washed in buffer, post fixed in 1% OsO_4_ in the same buffer for 2 hours. After dehydration with graded ethanol series followed by propylene oxide, they were embedded in Epon. Ultrathin sections were prepared with a Leica ultracut E microtome, stained with Uranyl acetate and lead citrate and observed with a JEOL 1200E electron microscope. Immuno-electron microscopy on ultrathin cryosections was performed as described elsewhere [68] using anti-GFP antibodies on GFP-TgAtg8 transfected parasites.

### Plaque assays

Confluent monolayers of HFF grown in 6-well plates were infected with ∼50 tachyzoites per well and incubated for 6–7 days at 37°C. They were then fixed in cold methanol for 20 minutes and stained with Giemsa stain. Images were obtained with an Olympus MVX10 macro zoom microscope equipped with an Olympus XC50 camera. Plaque area measurements were performed with Cell^A^ software (Olympus).

### Gene accession numbers

*TgAtg8* (TGME49_054120, http://toxodb.org); *TgAtg3* (TGME49_036110, http://toxodb.org)

## Supporting Information

Figure S1 **Immunolocalisation of TgAtg8 in extracellular tachyzoites.** Immunofluorescence analysis of parental RHΔHX (A) and transgenic GFP-TgAtg8 (B) extracellular parasites using anti-TgAtg8 antibody. Vesicular signal (arrowheads) was detected in addition to a cytosolic localisation.
(TIF)

Figure S2 **GFP-TgAtg8 vesicular signal.** A. GFP-TgAtg8-expressing extracellular tachyzoites were starved for amino acids during increasing periods of time and, in parasites displaying a punctate GFP signal, the numbers of puncta were counted. Results shown are means from 3 independent experiments ± SD. B. Intracellular (top) and extracellular (bottom) tachyzoites displaying a main punctate GFP-TgAtg8 signal in the apical region, as shown by localisation next to the apicoplast revealed with α–ACP antibody.
(TIF)

Figure S3 **Amino acid alignment of the C-terminal end of predicted Atg8 proteins from selected eukaryotic species.** The arrow shows the glycine residue used for lipidation. Sequences were retrieved from genomic databases (www.apidb.org, www.genedb.org, GenBank) and are as follows: *Toxoplasma gondii* (*T. gondii*, TGME49_054120), *Neospora caninum* (*N. caninum*, NC_LIV_041240), *Eimeria tenella* (*E. tenella*, TWINSCAN_PHASES00000233445), *Theleria annulata* (*T. annulata*, TA12615), *Plasmodium berghei* (*P. berghei*, PB000658.01.0), *P. chabaudi* (PC000787.01.0), *P. knowlesi* (PKH_060390), *P. vivax* (Pv001860), *P. falciparum* (PF10_0193), *Cryptosporidium muris* (*C. muris*, CMU_014110), *C. hominis* (Chro.70444), *C. parvum* (cgd7_3990), *Leishmania major* (*L. major*, LmjF19.1630), *S. cerevisiae* (NP_009475), *A. thaliana* (NP_001078424), *Schistosoma mansoni* (*S. mansoni*, 29295.t000044), *Danio rerio* (*D. rerio*, BAF43578.1), *Homo sapiens* (*H. sapiens*, NP_852610.1). Sequences were aligned using the MUSCLE algorithm. Color scale shows the degree of amino acid conservation between sequences.
(TIF)

Figure S4 **Separation of soluble and membrane-bound form of native TgAtg8.** Cell extracts from TgAtg8 parasites were subjected to a centrifugation at 100,000 g to separate a soluble fraction (high speed supernatant, HSS) from a membrane fraction (high speed pellet, HSP). The faster migrating form of TgAtg8 is exclusively present in the membrane fraction as revealed by Western blot analysis after urea SDS-PAGE using anti-TgAtg8.
(TIF)

Figure S5 **Some GFP-TgAtg8 signal co-localises partially with the Golgi apparatus in dividing tachyzoites.** GFP-TgAtg8 (top) or mutant version GFP-TgAtg8-G/A (unable to bind autophagosomes, bottom) and RFP-tagged Golgi marker GRASP were imaged simultaneously in dividing parasites.
(TIF)

Figure S6 **Effects of kinase inhibitors on the modulation of autophagy in extracellular tachyzoites.** A. Extracellular tachyzoites expressing GFP-TgAtg8 were incubated in the presence or not of rapamycin, the inhibitor of TOR kinase, for 8 hours in complete DMEM medium with 10% serum. The proportions of cells displaying punctate (right) or cytosolic (left) GFP-TgAtg8 signals were assessed. Data are mean from *n* = 3 independent experiments ±SEM. B. Extracellular tachyzoites expressing GFP-TgAtg8 were incubated in the presence or not of wortmannin and 3-methyladenine, two inhibitors of the PI3K, for 8 hours in starvation medium (HBSS). The proportions of cells displaying punctate or cytosolic GFP-TgAtg8 signals were assessed. T0: control at the start of the experiment; T8: timepoint after 8 hours in HBSS. Data are mean from *n* = 3 independent experiments ±SEM. (* p<0.05, ** p<0.005, Student's T test).
(TIF)

Figure S7 **Alignment of amino acid sequences from yeast (YNR007C), human (AAH24221) and** ***Toxoplasma*** **Atg3 orthologues using the MUSCLE algorithm.** Second putative starting methionine for the *Toxoplasma* sequence is indicated by an arrow. The red asterisk denotes the active site cysteine.
(TIF)

Figure S8 **Depletion of TgAtg3 does not appear to modify several of the subcellular organelles in tachyzoites.** Intracellular tachyzoites were analysed by immunofluorescencence for apicoplast (A, in red), IMC (B, in green), micronemes (C, in green) and rhoptries (D, in green) markers (see materials and methods for details). Efficient depletion of inducible TgAtg3 copy was checked by anti-myc labelling. DAPI staining of the DNA was also shown when available.
(TIF)

Figure S9 **Loss of mitochondrial membrane potential in TgAtg3-depleted parasites.** Mitochondrial membrane potential was detected using Mitotracker Red CMXRos labelling in conditional TgAtg3 KO parasites either untreated, or treated for 2 or 4 days with ATc, and co-labelled with a mitochondrial protein marker (see materials and methods for details). TgAtg3-expressing cell line imyc-sTgAtg3 incubated for 4 days in the presence of ATc was used as a control.
(TIF)

Figure S10 **Mitochondrion fragmentation is not an early event of tachyzoite cell death.** Intracellular parasites were treated for up to 2 days with 1 µM pyrimethamine to induce cell death and checked by immunofluorescence with mitochondrial and micronemal markers (see materials and methods for details). Tachyzoites displayed loss of morphology and abnormal distribution of micronemes before fragmentation of the mitochondrion. The series of micrographs in the middle were taken after 1 day of pyrimethamine treatment. The series of micrographs at the bottom were taken after 2 days of treatment and show partial fragmentation of the mitochondrial network (arrows), while it is still intact in other cells (arrowhead).
(TIF)

Table S1 **Orthologues of Atg proteins found in the genome of** ***T. gondii*** **.** The core machinery for autophagosome formation appears conserved in *T. gondii* (in red), while the Atg5-Atg12 conjugation pathway is poorly conserved (blue). Yeast Atg proteins were searched with BLAST against *T. gondii* genomic database (www.toxodb.org); E value and percentage of pairwise identity with yeast were reported when a putative orthologue was found; nd: not detected. Successful reverse BLAST search against NCBI database (www.ncbi.nlm.nih.gov/blast/), when retrieving the Atg orthologue from another genus, is indicated. Presence of orthologues in other apicomplexan genomes was checked by BLAST search in the www.eupathdb.org and www.genedb.org databases. Nc: *Neospora caninum*, Pf: *Plasmodium Falciparum*, Cp: *Cryptosporidium parvum*, Et: *Eimeria tenella*, Ta: *Theileria annulata*.
(XLS)

Table S2 **Primers used in this study.** Restriction sites used for cloning were underlined.
(XLS)
