# Supplementary figures and images for: Autophagy Protein Atg3 is Essential for Maintaining Mitochondrial Integrity and for Normal Intracellular Development of Toxoplasma gondii Tachyzoites
Source: PLoS Pathog. 2011 Dec 1;7(12):e1002416. doi: 10.1371/journal.ppat.1002416 (PMC3228817; doi:10.1371/journal.ppat.1002416)

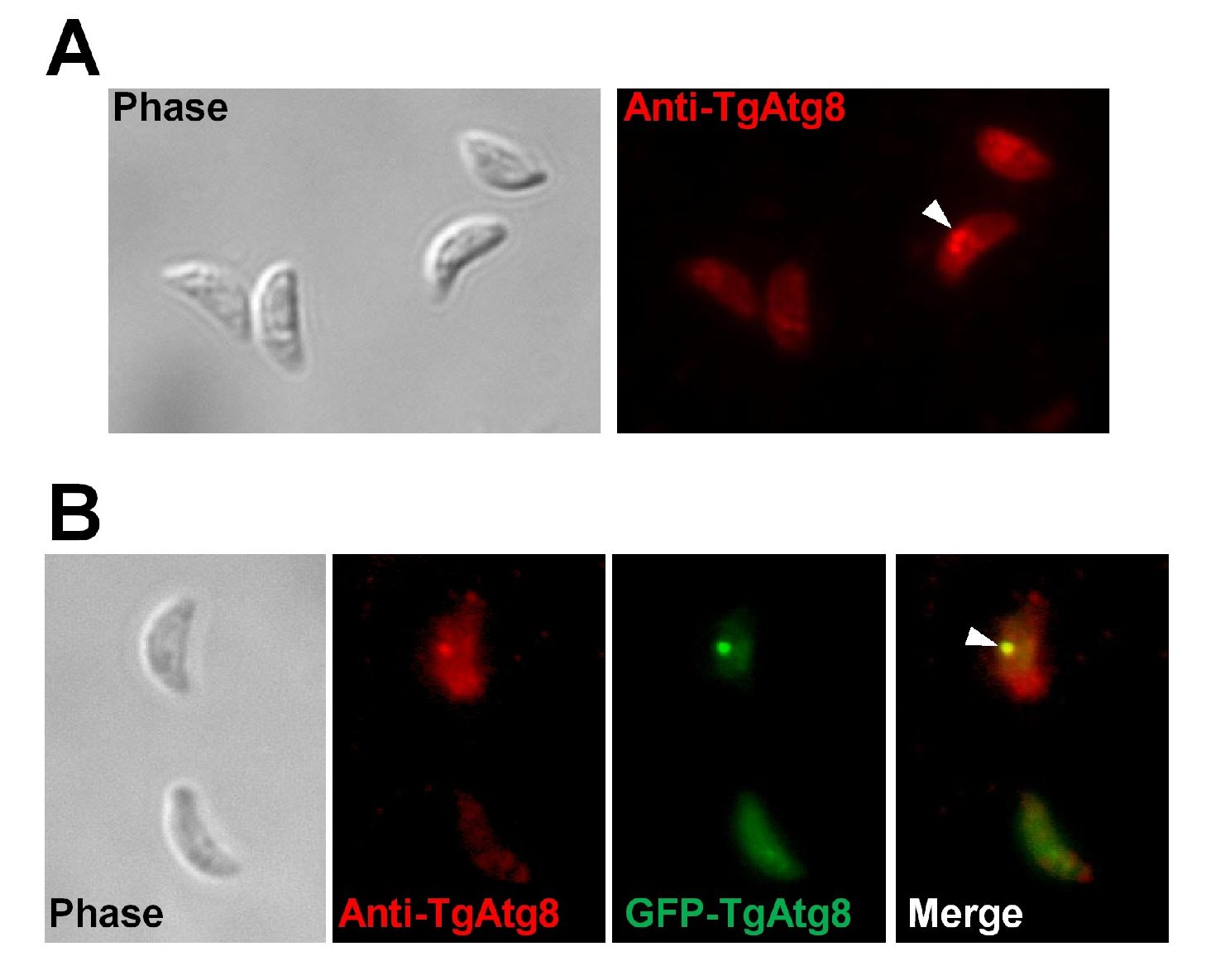

Supplement: Figure S1 — Immunolocalisation of TgAtg8 in extracellular tachyzoites. Immunofluorescence analysis of parental RHΔHX (A) and transgenic GFP-TgAtg8 (B) extracellular parasites using anti-TgAtg8 antibody. Vesicular signal (arrowheads) was detected in addition to a cytosolic localisation. (TIF) [file ppat.1002416.s001.tif]

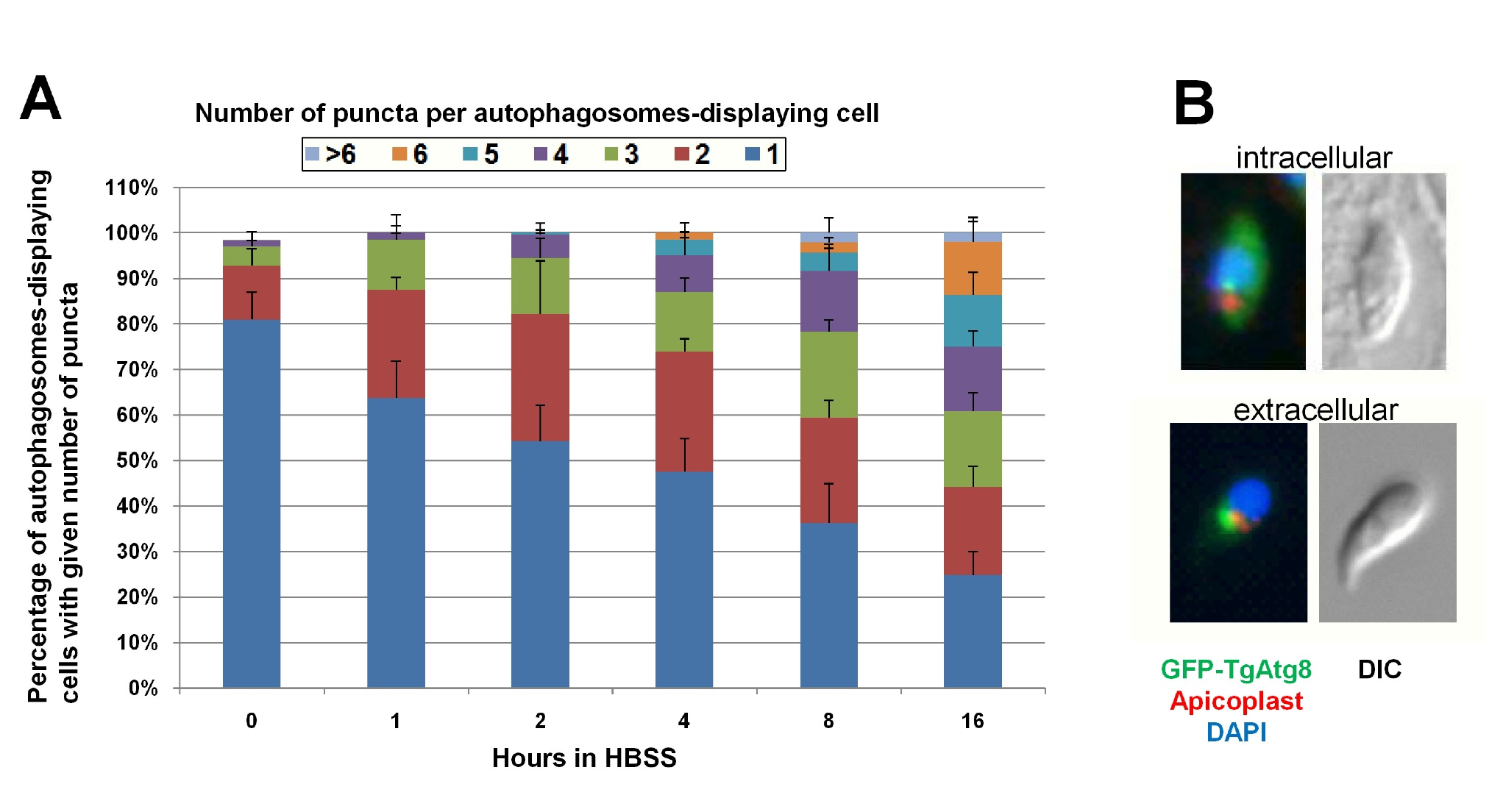

Supplement: Figure S2 — GFP-TgAtg8 vesicular signal. A. GFP-TgAtg8-expressing extracellular tachyzoites were starved for amino acids during increasing periods of time and, in parasites displaying a punctate GFP signal, the numbers of puncta were counted. Results shown are means from 3 independent experiments ± SD. B. Intracellular (top) and extracellular (bottom) tachyzoites displaying a main punctate GFP-TgAtg8 signal in the apical region, as shown by localisation next to the apicoplast revealed with α–ACP antibody. (TIF) [file ppat.1002416.s002.tif]

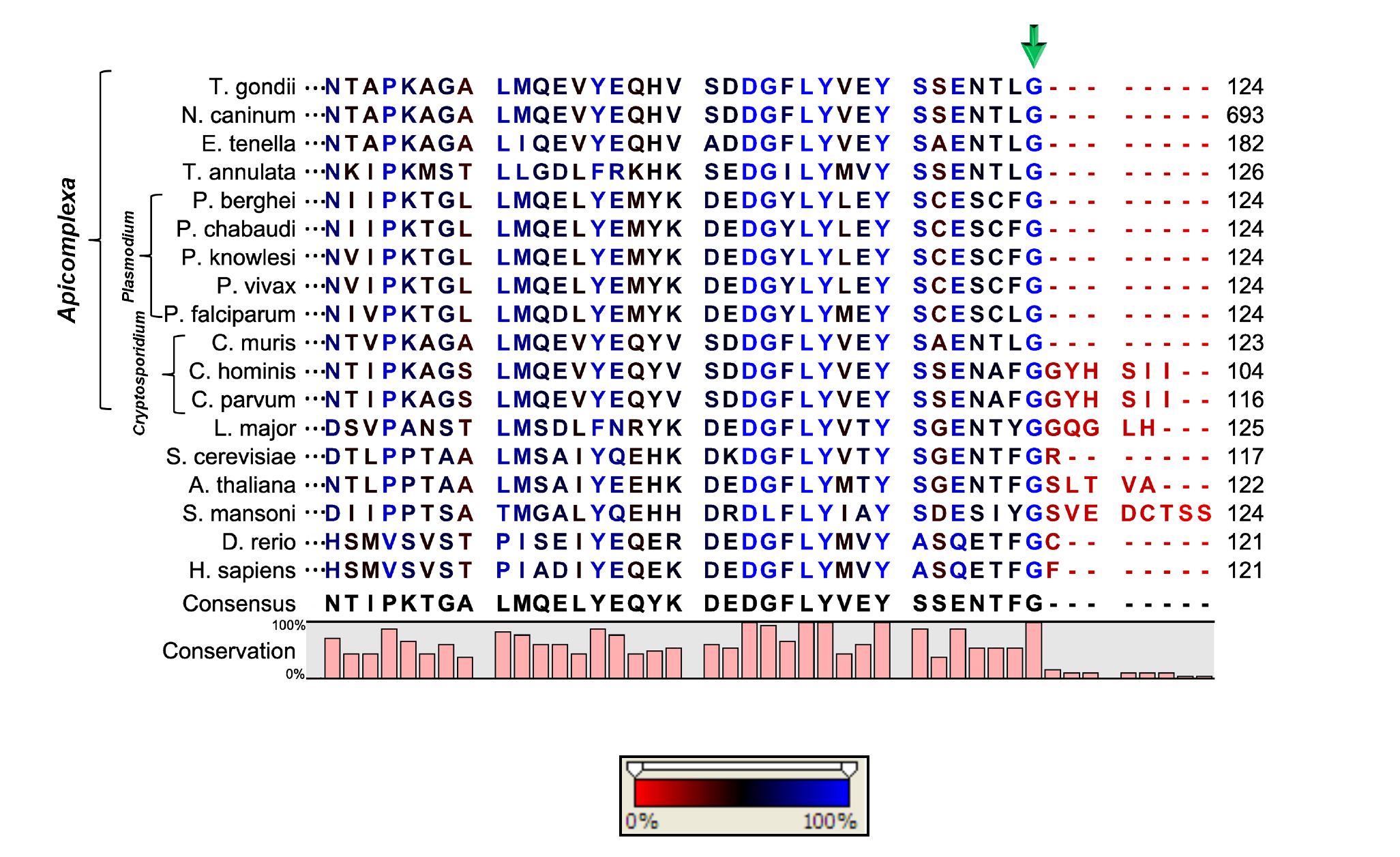

Supplement: Figure S3 — Amino acid alignment of the C-terminal end of predicted Atg8 proteins from selected eukaryotic species. The arrow shows the glycine residue used for lipidation. Sequences were retrieved from genomic databases (www.apidb.org, www.genedb.org, GenBank) and are as follows: Toxoplasma gondii (T. gondii, TGME49_054120), Neospora caninum (N. caninum, NC_LIV_041240), Eimeria tenella (E. tenella, TWINSCAN_PHASES00000233445), Theleria annulata (T. annulata, TA12615), Plasmodium berghei (P. berghei, PB000658.01.0), P. chabaudi (PC000787.01.0), P. knowlesi (PKH_060390), P. vivax (Pv001860), P. falciparum (PF10_0193), Cryptosporidium muris (C. muris, CMU_014110), C. hominis (Chro.70444), C. parvum (cgd7_3990), Leishmania major (L. major, LmjF19.1630), S. cerevisiae (NP_009475), A. thaliana (NP_001078424), Schistosoma mansoni (S. mansoni, 29295.t000044), Danio rerio (D. rerio, BAF43578.1), Homo sapiens (H. sapiens, NP_852610.1). Sequences were aligned using the MUSCLE algorithm. Color scale shows the degree of amino acid conservation between sequences. (TIF) [file ppat.1002416.s003.tif]

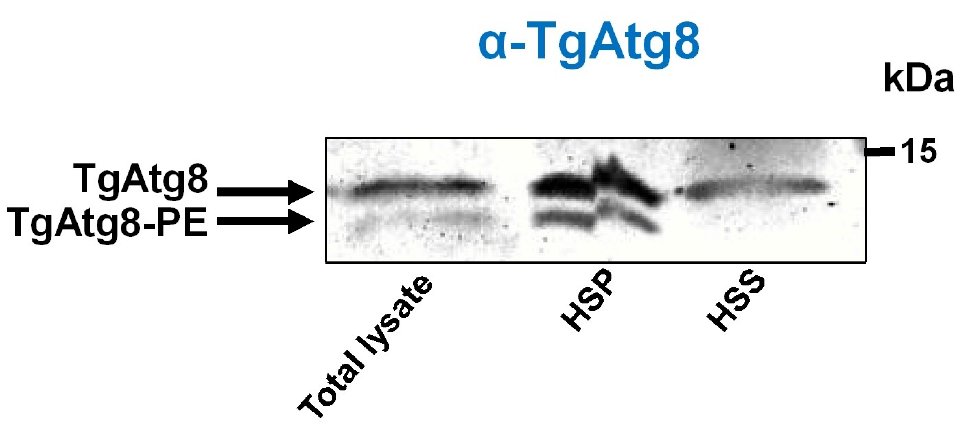

Supplement: Figure S4 — Separation of soluble and membrane-bound form of native TgAtg8. Cell extracts from TgAtg8 parasites were subjected to a centrifugation at 100,000 g to separate a soluble fraction (high speed supernatant, HSS) from a membrane fraction (high speed pellet, HSP). The faster migrating form of TgAtg8 is exclusively present in the membrane fraction as revealed by Western blot analysis after urea SDS-PAGE using anti-TgAtg8. (TIF) [file ppat.1002416.s004.tif]

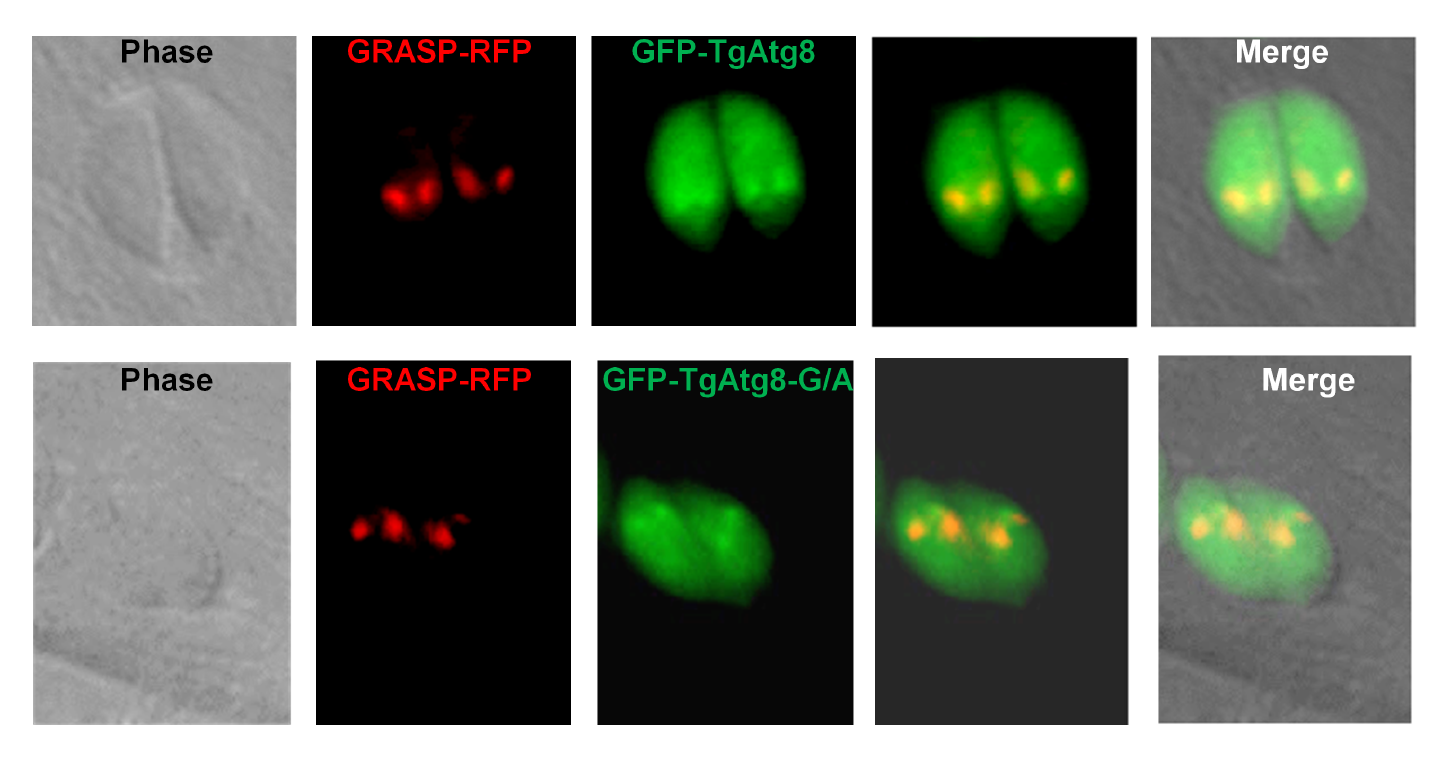

Supplement: Figure S5 — Some GFP-TgAtg8 signal co-localises partially with the Golgi apparatus in dividing tachyzoites. GFP-TgAtg8 (top) or mutant version GFP-TgAtg8-G/A (unable to bind autophagosomes, bottom) and RFP-tagged Golgi marker GRASP were imaged simultaneously in dividing parasites. (TIF) [file ppat.1002416.s005.tif]

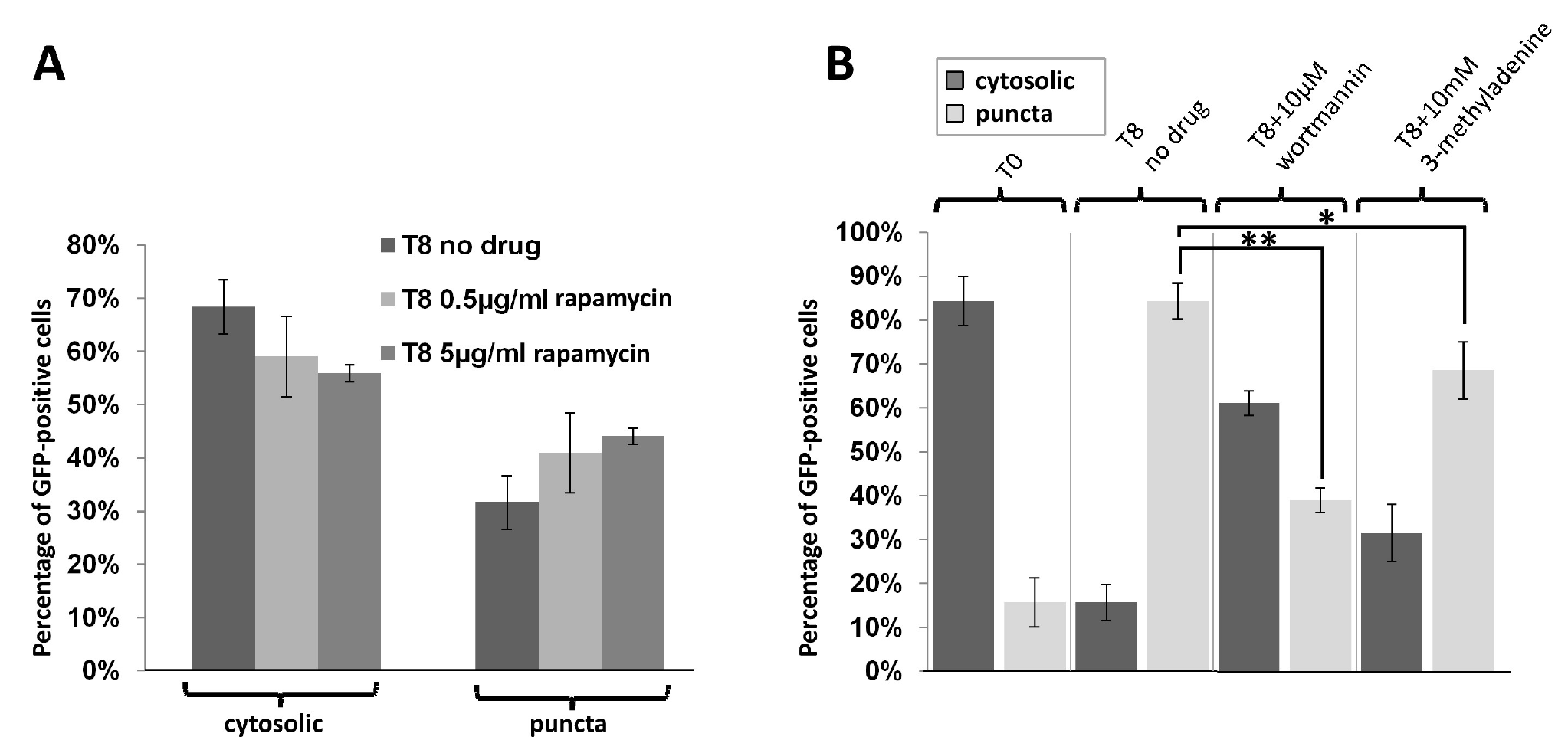

Supplement: Figure S6 — Effects of kinase inhibitors on the modulation of autophagy in extracellular tachyzoites. A. Extracellular tachyzoites expressing GFP-TgAtg8 were incubated in the presence or not of rapamycin, the inhibitor of TOR kinase, for 8 hours in complete DMEM medium with 10% serum. The proportions of cells displaying punctate (right) or cytosolic (left) GFP-TgAtg8 signals were assessed. Data are mean from n = 3 independent experiments ±SEM. B. Extracellular tachyzoites expressing GFP-TgAtg8 were incubated in the presence or not of wortmannin and 3-methyladenine, two inhibitors of the PI3K, for 8 hours in starvation medium (HBSS). The proportions of cells displaying punctate or cytosolic GFP-TgAtg8 signals were assessed. T0: control at the start of the experiment; T8: timepoint after 8 hours in HBSS. Data are mean from n = 3 independent experiments ±SEM. (* p<0.05, ** p<0.005, Student's T test). (TIF) [file ppat.1002416.s006.tif]

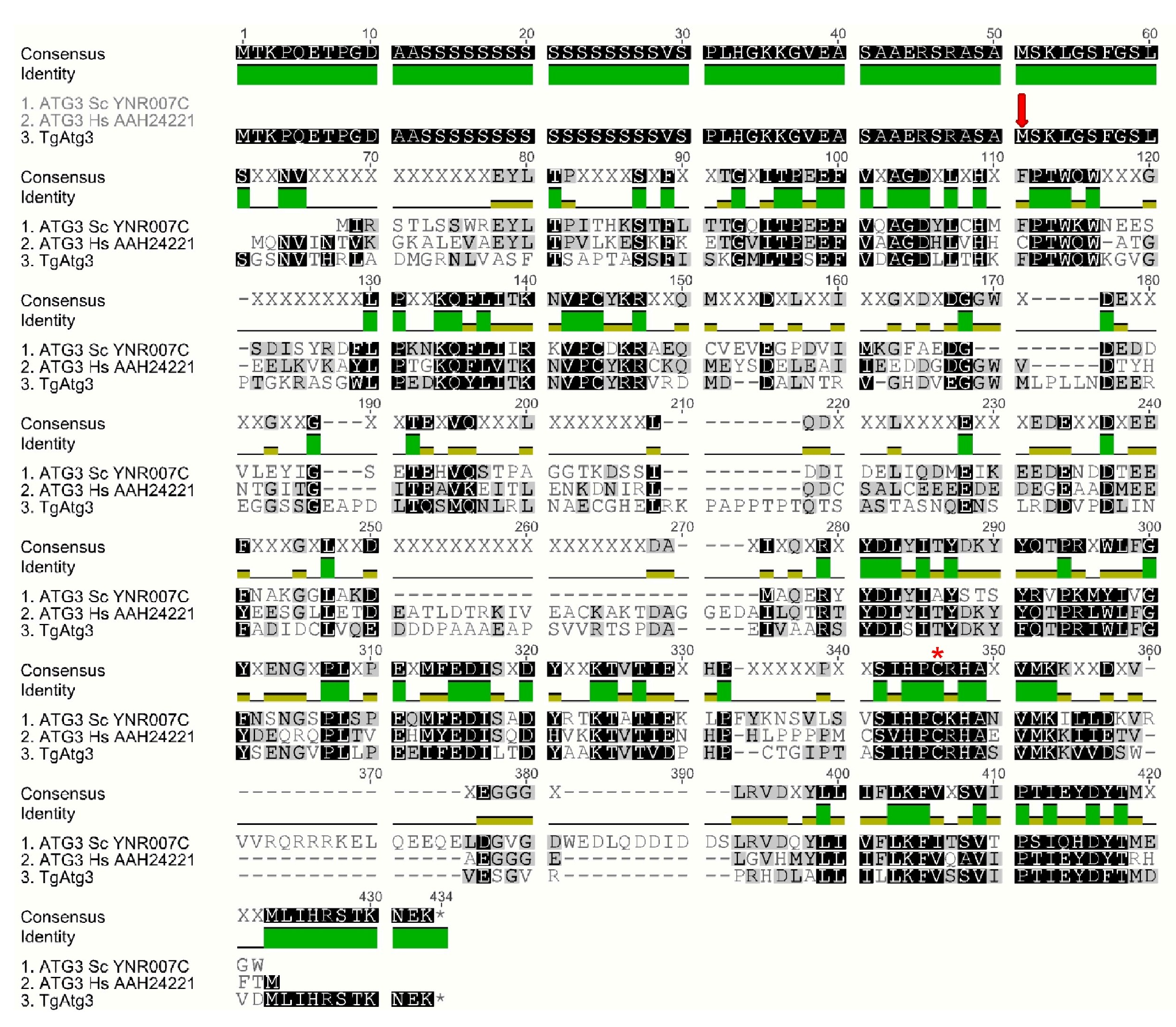

Supplement: Figure S7 — Alignment of amino acid sequences from yeast (YNR007C), human (AAH24221) and Toxoplasma Atg3 orthologues using the MUSCLE algorithm. Second putative starting methionine for the Toxoplasma sequence is indicated by an arrow. The red asterisk denotes the active site cysteine. (TIF) [file ppat.1002416.s007.tif]

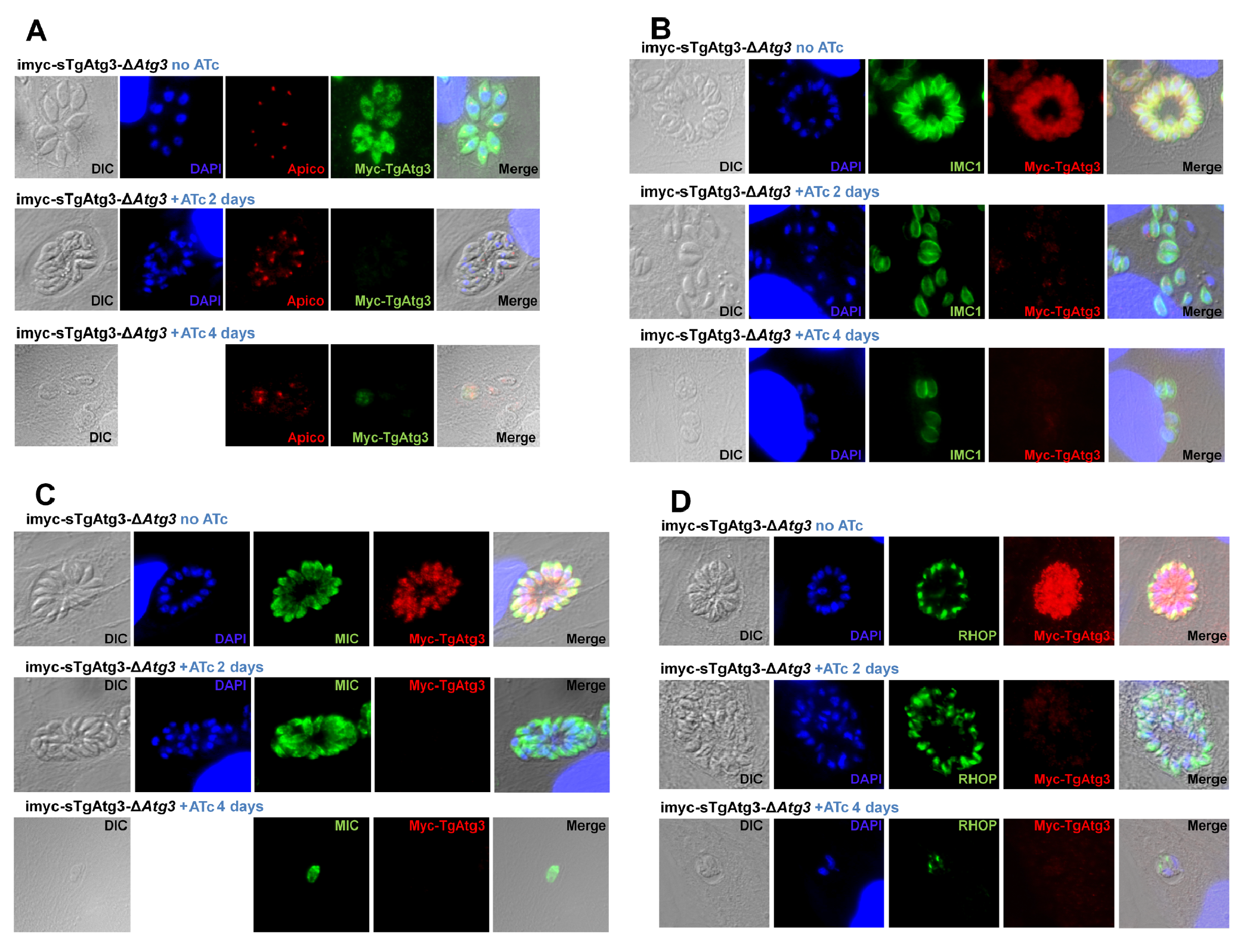

Supplement: Figure S8 — Depletion of TgAtg3 does not appear to modify several of the subcellular organelles in tachyzoites. Intracellular tachyzoites were analysed by immunofluorescencence for apicoplast (A, in red), IMC (B, in green), micronemes (C, in green) and rhoptries (D, in green) markers (see materials and methods for details). Efficient depletion of inducible TgAtg3 copy was checked by anti-myc labelling. DAPI staining of the DNA was also shown when available. (TIF) [file ppat.1002416.s008.tif]

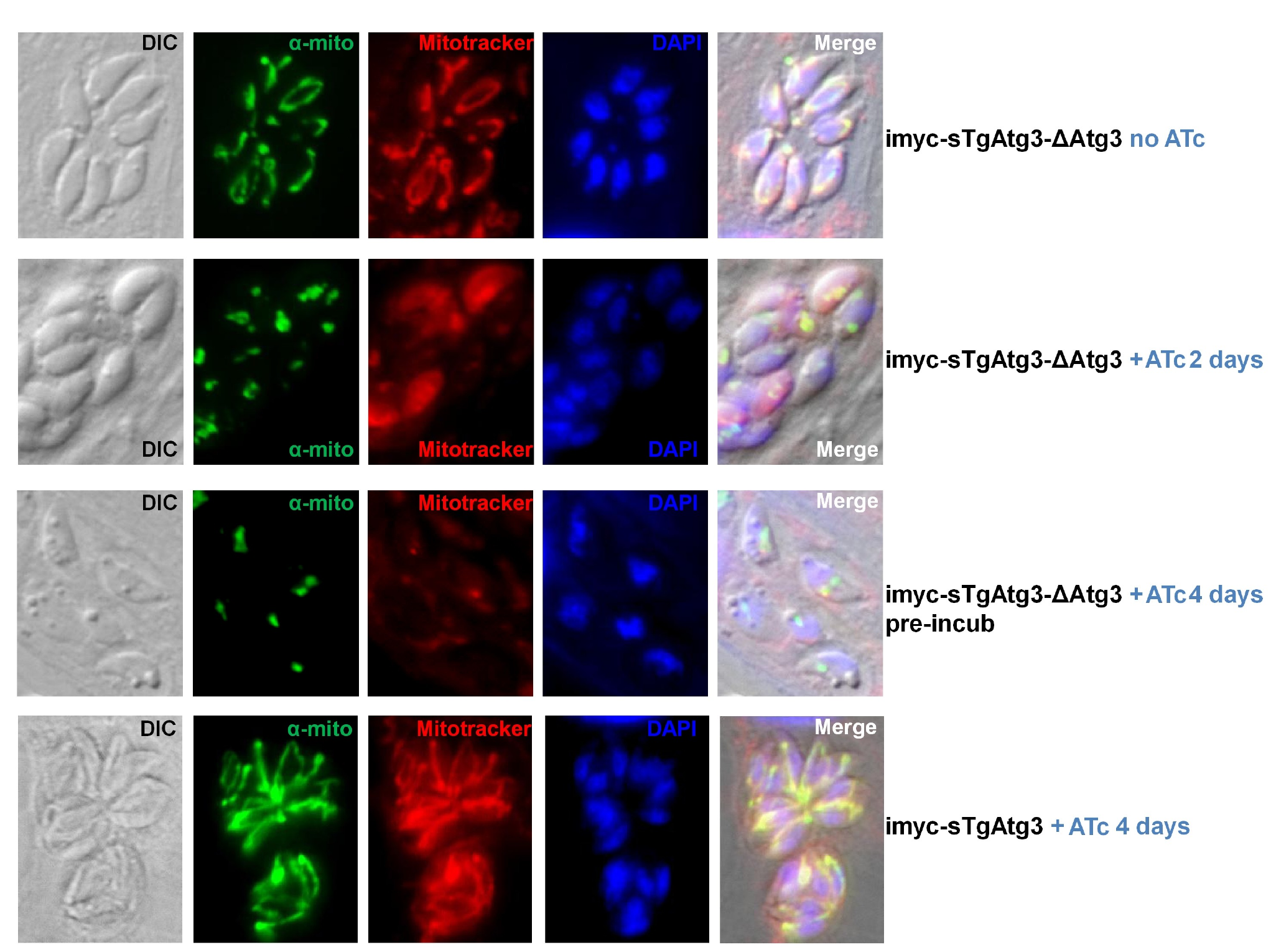

Supplement: Figure S9 — Loss of mitochondrial membrane potential in TgAtg3-depleted parasites. Mitochondrial membrane potential was detected using Mitotracker Red CMXRos labelling in conditional TgAtg3 KO parasites either untreated, or treated for 2 or 4 days with ATc, and co-labelled with a mitochondrial protein marker (see materials and methods for details). TgAtg3-expressing cell line imyc-sTgAtg3 incubated for 4 days in the presence of ATc was used as a control. (TIF) [file ppat.1002416.s009.tif]

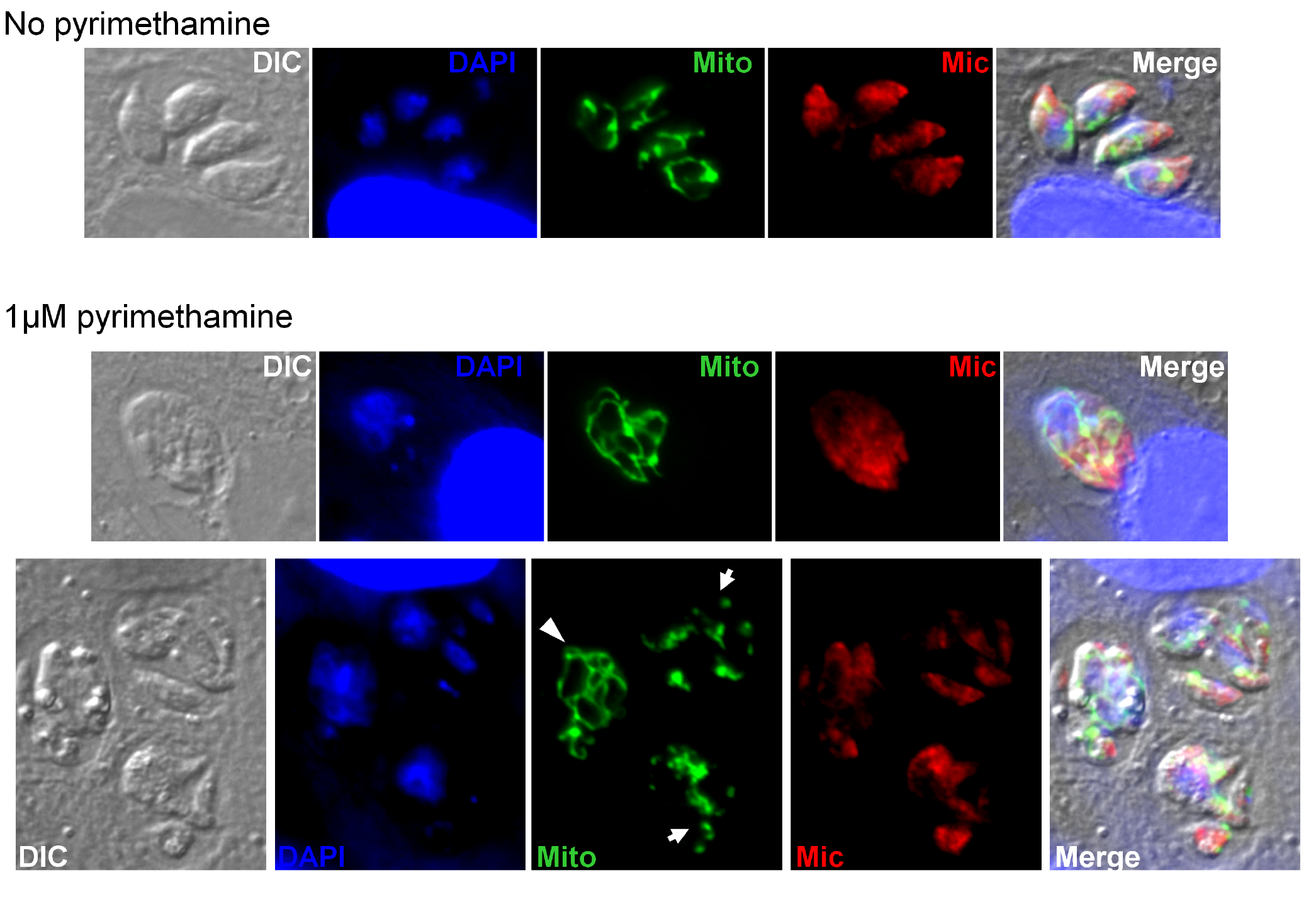

Supplement: Figure S10 — Mitochondrion fragmentation is not an early event of tachyzoite cell death. Intracellular parasites were treated for up to 2 days with 1 µM pyrimethamine to induce cell death and checked by immunofluorescence with mitochondrial and micronemal markers (see materials and methods for details). Tachyzoites displayed loss of morphology and abnormal distribution of micronemes before fragmentation of the mitochondrion. The series of micrographs in the middle were taken after 1 day of pyrimethamine treatment. The series of micrographs at the bottom were taken after 2 days of treatment and show partial fragmentation of the mitochondrial network (arrows), while it is still intact in other cells (arrowhead). (TIF) [file ppat.1002416.s010.tif]
